# Development of a 3D-Printed Photometric Device for the Determination of Urea in Milk Samples

**DOI:** 10.1021/acsomega.5c09412

**Published:** 2026-05-21

**Authors:** Natália dos Santos Trindade, Saidy Cristina Ayala-Durán, Gabriel Baroffaldi Piassalonga, João Pedro Silva, Eduardo Luiz Rossini, Maria Valnice Boldrin Zanoni, Paulo Clairmont Feitosa de Lima Gomes

**Affiliations:** Sao Paulo State University (UNESP), Institute of Chemistry, Department of Analytical Chemistry, Physical Chemistry and Inorganic Chemistry, National Institute for Alternative Technologies of Detection, Toxicological Evaluation and Removal of Micropollutants and Radioactives (INCT-DATREM), 28108 São Paulo State University (UNESP), Araraquara, São Paulo 14800-060, Brazil

## Abstract

This work reports the development and application of a user-friendly, portable, and low-cost 3D-printed photometer for the detection of urea adulteration in milk. The method is based on a colorimetric reaction with *p*-dimethylaminocinnamaldehyde combined with a rapid (30 min), economical, and environmentally friendly sample preparation involving calcium chloride salting-out and acidification (pH 3.8), forming a chromophore with maximum absorption at 530 nm. The device demonstrated a linear range of 4.0–40 mg mL^–1^, a limit of quantification of 4.0 mg mL^–1^, recoveries of 97–105%, and intra- and interday precision between 1.43% and 3.39% in whole and skim milk. Results were statistically comparable (*p* > 0.05) to a benchtop spectrophotometer and HPLC-UV–vis. Released as open-source hardware under CC BY-SA 4.0, this analytical platform combines low-cost fabrication, modular electronics, and wireless capabilities to provide decentralized, accessible, and high-accuracy milk quality monitoring, thereby strengthening food safety while reducing analytical costs and environmental impact.

## Graphical abstract

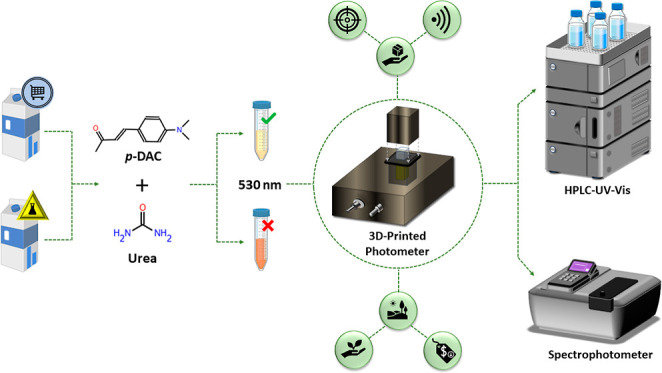

## 1 Introduction

Milk contamination can occur during dairy production, involving biological and/or chemical processes.^1–4^ Biological contamination is related to inadequate milking equipment practices.^5^ Chemical contamination can be caused by the use and consumption of veterinary products (legal or illegal), pesticides, agrochemicals, contaminated forage and feed, or by the improper use of chemical compounds during the stages of milk production, processing, packaging, and storage.^6^

Among the adulterants commonly used in milk are water, salt, sugar, hydrogen peroxide, and synthetic urea.^7^ The addition of urea is an adulteration practice used to mask dilution with water, artificially increase the final volume of the product, and alter the proportions of nonprotein nitrogenous compounds. This practice also reduces titratable acidity and inhibits milk fermentation.^8^ Although urea may be naturally present in raw milk due to its heterogeneous composition, it is frequently added to restore the standard protein percentage required for consumption.^9^

According to the Food Safety and Standards Authority of India (FSSAI), the maximum permitted level is 0.7 mg mL^–1^, as established by the Prevention of Food Adulteration (PFA) Act of 2006.^7^ In Brazil, Normative Instructions No. 76 and 77 of the Ministry of Agriculture, Livestock and Supply,^10^ prohibit the presence of foreign substances in refrigerated raw milk and require rigorous quality control throughout the production chain. In the European Union, Regulation (EU) 605/2010 ensures the traceability and safety of milk and dairy products, requiring registration, monitoring, and prohibiting the addition of foreign substances.^11^ In the United States, while no specific maximum limit for urea in milk is defined, the Food and Drug Administration regulates milk quality under the Federal Food, Drug, and Cosmetic Act and the Pasteurized Milk Ordinance, which prohibit the addition of foreign or economically motivated adulterants and consider excessive nonprotein nitrogen compounds, such as urea, as evidence of milk adulteration.^12,13^

Traditional and advanced analytical methods for determining milk quality and urea fraud are based on nitrogen quantification, titration, chromatography, enzymatic and spectrophotometry, as well as the use of biosensors.^14–16^ These approaches involve specific, robust, and nonportable equipment, require trained and skilled analysts, and increase the cost per analysis.

The Kjeldahl method, established by the Association of Official Analytical Chemists (AOAC)^17^ is the official procedure for determining organic nitrogen in food matrices such as milk. Although accurate, it is a complex and time-consuming method that involves acid digestion, alkaline distillation to release ammonia, and subsequent titration.^18^ In turn, the enzymatic method is based on the hydrolysis of urea by the urease enzyme, followed by quantification of the ammonium produced.^19^ However, this method generally requires constant monitoring of urease activity, strict pH control, measurement of ammonia formed, and prevention of enzymatic denaturation.

In the context of developing clean analytical methods, colorimetric methods have been developed for the determination of urea in milk, using chromogenic reagents such as diacetyl monoxime, *p*-dimethylamine cinnamaldehyde (*p*-DAC), and *p*-dimethylamine benzaldehyde (*p*-DMAB).^20–23^ The use of diacetyl monoxime as a chromophore requires sulfuric acid, phosphoric acid, thiosemicarbazide, and ferric chloride, making the method less clean and operationally complex.^24^ In contrast, the use of the chromogenic reagents *p*-DAC and *p*-DMAB,^25,26^ offers advantages such as simplicity, low reagent consumption and waste, and good reaction stability, with *p*-DAC being less toxic and harmful compared to *p*-DMAB. Additionally, it provides rapid, reliable results, making it a clean, environmentally sustainable approach.

Thus, developing simple, portable, and affordable devices that enable easy data acquisition and yield results comparable to those of conventional instruments is essential for advancing chemical analyses for quality control, particularly for perishable samples such as milk.^27^ The low cost and design flexibility offered by additive manufacturing further support the development of efficient analytical devices, as rapid adjustments and modifications can be made during the design process. This has contributed to the increasing adoption of 3D printing in analytical chemistry, as reported in numerous studies.^28–30^

Devices manufactured through material extrusion are produced by converting a virtual model, designed in computer-aided design (CAD) software, into a signal compatible with 3D printers. The fused deposition modeling (FDM) process uses thermoplastic filaments that are melted in the printer’s extruder nozzle and deposited layer by layer, enabling the creation of customized objects tailored to the user’s needs. The integration of 3D printing with technologies such as ESP/Arduino microcontrollers and electronic components, including LEDs, photoresistors, and resistors, combined with additive manufacturing, enables the development of low-cost, portable devices for chemical analysis that provide rapid, accurate results.^27^

Recently, Baumgartner et al.^31^ presented a device compatible with commercially available benchtop spectrometers. The module was printed using PLA (polylactic acid) filament, with a total system cost of 360–600 EUR. The proposed system demonstrated potential for applications requiring high sample throughput or handling complex, potentially biohazardous samples, including samples containing *Escherichia coli* and bovine serum albumin.

Nandeshwar et al.^27^ developed a portable colorimetric sensor for urea detection in milk using *p*-DMAB as the chromogenic reagent, with wireless data transmission to a smartphone via a Bluetooth-enabled Raspberry Pi RP2020 microcontroller. Although the system demonstrated the feasibility of low-cost optical detection, it exhibited limited linearity (*R* ^2^ = 0.720) over a narrow concentration range (0.1–0.7 mg mL^–1^), and neither the reaction mechanism nor the sample preparation procedure was systematically investigated. Analytical validation across different milk matrices was not reported. Moreover, *p*-DMAB requires more toxic reagents compared to *p*-DAC, raising concerns about reagent safety and environmental impact.

In the present work, we address these limitations by developing a portable 3D-printed photometer for the quantification of urea in milk using the safer chromophore *p*-DAC. Unlike prior work, this study provides, for the first time, a complete analytical framework. The reaction mechanism was elucidated by mass spectrometry, and the sample preparation based on CaCl_2_·2H_2_O salting-out was systematically optimized to effectively remove interfering substances. The method was fully validated across multiple UHT milk types with varying fat content (whole and skim milk), achieving a wide linear range (4.0–40 mg mL^–1^), recoveries of 97–105%, and intra- and interday precision below 5%. Results were statistically comparable (*p* > 0.05) to those obtained with spectrophotometric and HPLC-UV–Vis methods. The resulting platform combines low cost, modular open-source design, ease of fabrication, and portability, offering a practical and reliable solution for decentralized milk quality monitoring in field and resource-limited settings.

## 2 Materials and Methods

### 2.1 Fabrication of the 3D-Printed Photometer

The 3D-photometric device comprises multiple 3D-printable parts, an electronic circuit, a microcontroller board, a disposable cuvette (1 cm path), an LED as the light source, and a photodiode as the detector. All 3D-printable parts (housing, lid, and cuvette cover and base) were designed using Autodesk Inventor Professional 2024 software and printed by a Creality K1C printer, using ABS filament, extrusion temperature set at 260 °C, bed temperature at 100 °C, 270 mm s^–1^ printing speed, and 0.20 mm layer height (Figure 1).

**Figure 1:**
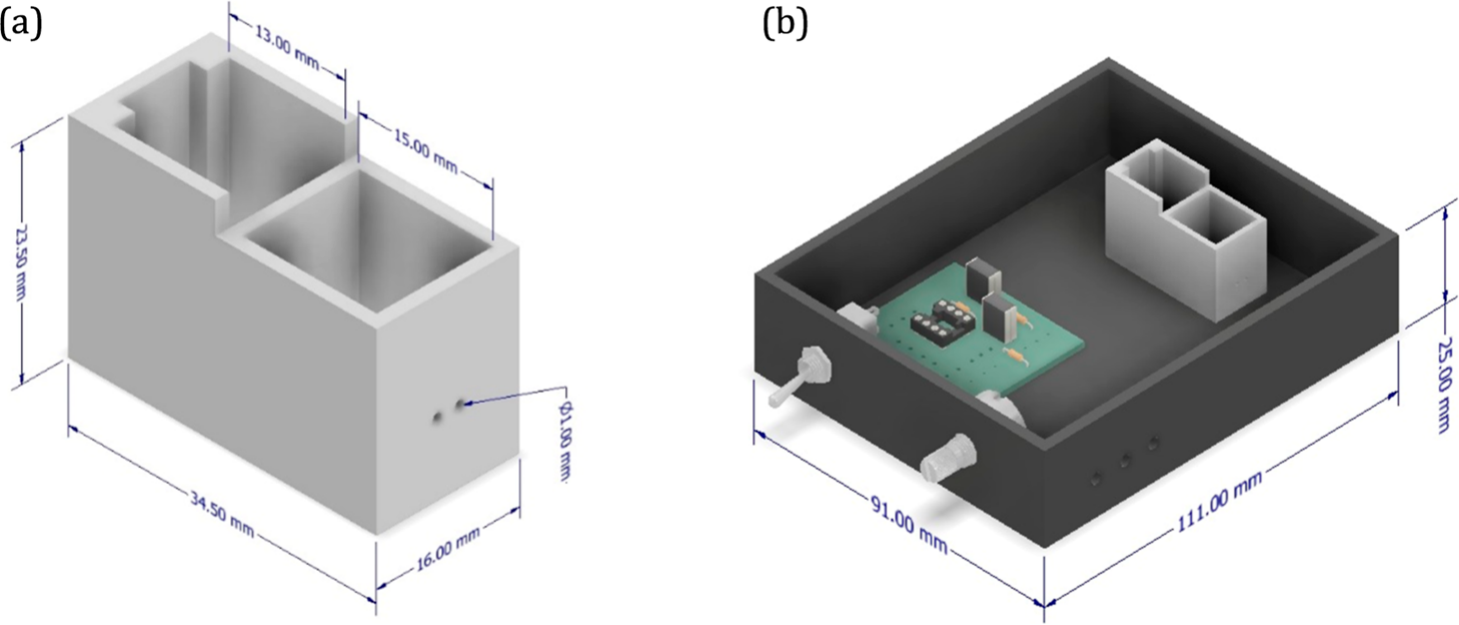
(a) Cuvette and the irradiation source compartments of the 3D-printed photometer, (b) 3D-photometer design in Inventor software.

#### 2.1.1 Design and Assembly of the 3D-Printed Photometer

As a light source, a high-brightness green LED (5 mm diameter) with a band emission of 520 ± 30 nm was used, as determined by a spectrometer (Ocean Optics model Red Tide USB 650; Figure S1). A BPW34 photodiode was used for detection, aligned at 180° to the light source. To amplify the low current from the photodiode, a transimpedance amplifier was required. A schematic electric circuit drawing, created using the KiCAD software, is presented in Figure 2a. The switch SWA1 allows for the selection of the gain of the circuit, where the resistor (R1 and R2) determines the amount of gain. At the same time, the capacitors (C1 and C2) filter electrical noise generated by signal amplification and the electronic circuit components (the low-pass filter). In this work, all photometric results were obtained with a gain provided by the 1 MΩ resistor (R1). The OPA344PA (U1) operational amplifier amplified the signal. The brightness of the LED (D3), used as a light source, is controlled by the electronic circuit shown in Figure 2a. The variable resistor (VR1) controls the potential drop across the LED and consequently its brightness.

**Figure 2:**
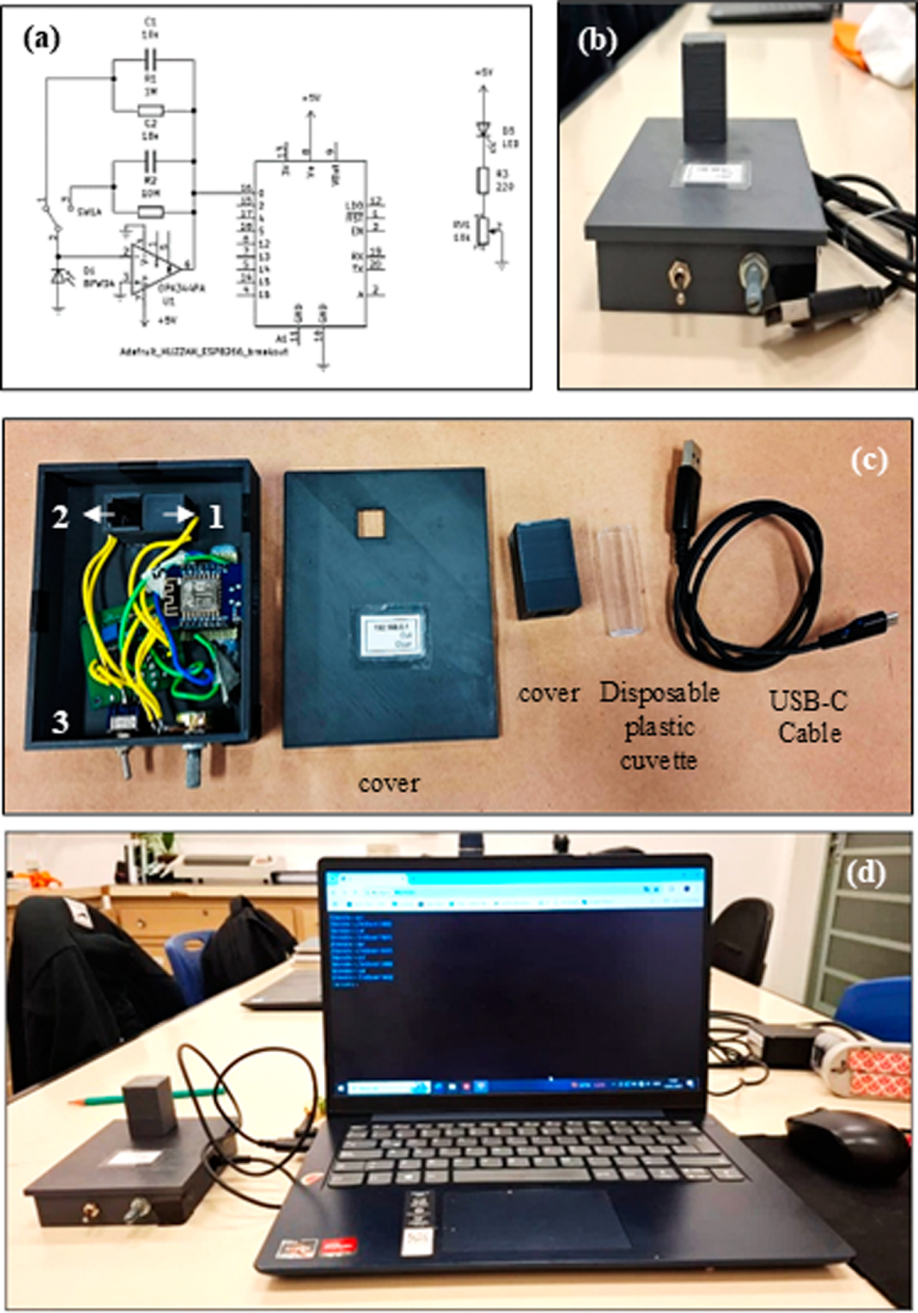
(a) Schematic drawing of an electrical circuit. (b) Frontal view photography of the 3D-printed photometer. (c) Parts that make up the 3D-printed photometer. (d) Photography of a 3D-printed photometer in operation during data acquisition.

According to the electrical schematic, a printed circuit board (PCB) for the amplification circuit was designed and manufactured by JLCPCB in China. The material chosen for construction was FR-4 Standard TG 135-140, with a board width of 1.6 mm and a HASL surface finish (including lead). The green LED and photodiode were wired to the PCB. The microcontroller board used for data acquisition and powering all circuits and components was the ESP8266 D1 mini. This board was equipped with an ESP8266EX microcontroller. All details about it can be found in its datasheet.^32^ The ESP8266 D1 mini has 11 digital input/output ports, 1 analog input port (0 - 3.2 V, 10-bit resolution), and 4 MB of data storage. It can connect multiple devices via Wi-Fi and Bluetooth. The 5.0 V, ground, and analog pins of the microcontroller board were wired to the PCB, connected to the first and second pins used to feed and close the circuit, and the third pin used to measure the operational amplifier’s output voltage.

The microcontroller was programmed in C++, written and compiled using the Arduino IDE. The program, running on a web server, implements a file system that uses the controlling board’s storage space and creates a Wi-Fi access point for client connections, allowing access to the device’s web server via a web browser. This web server has end points for retrieving the analog output in JSON format,^33^ downloading, and uploading HTML files. A command-line interface (CLI) and an HTML uploader program were created using HTML and JavaScript, respectively, both of which can access the web server to retrieve analog readings and upload HTML files. All software and related information are available in a Git repository at https://github.com/kpi-jps/photometer_cli, along with Supporting Information (Appendix A and Table S1). Figure 2b shows a photograph of the front view of the 3D-printed photometric device.

The device consisted of three main parts: a smaller closed compartment containing the LED source and the photodetector (Figure 2c). The second compartment, together with the base compartment, had space for the polystyrene (PS) cuvette, centered at the bottom of the device, aligned with the radiation beam and the detection compartments (Figure 2c). A small 1.0 mm hole was created between the cuvette and irradiation source compartments, which attenuates and focuses the light beam before it reaches the cuvette (Figure 1). The third, larger compartment (3) contained the electronic circuit, the microcontroller board, and wires (Figure 2c). The top cover allowed the device to be closed (Figure 2b), and a smaller cover closed the cuvette, preventing interference from external light during analysis (Figure 1b). All dimensions of photometer compartments are presented in Figure 1.

To use the device, a 5.0 V, 1 A power supply or a PC USB port can be used, as shown in Figure 2d. Detailed information about the setup process and device usage is provided in the Supporting Information. The generated analytical analog signal is converted to a digital signal and recorded by a microcontroller using its C++ program. Before outputting the result, it applies the moving average to the recorded signals and converts bits to millivolts.

The absorbance value for photometric measurements was determined using eq 1, where *S*_sample_ and *S*_blank_ correspond to the voltage signals (in millivolts) obtained for the sample and the blank, respectively.

1 $$absorbance=-\log\left(\frac{S_{sample}}{S_{blank}}\right)$$

#### 2.1.2 3D-Photometer Setup

As mentioned earlier, the photometric device is controlled by an ESP8266 D1 mini board. Before using the photometer, the C++ program presented in Section 2.1.1. Section 2.1.1 must be compiled and transferred to the microcontroller. This can be done using the Arduino IDE by including all board information in the “Boards manager” menu and all libraries used by the C++ programming sketch (ESP8266WiFi, FS, LittleFS) in the “Library manager” menu. To retain the feature that allows sending files to the controller board, such as an HTML user interface, choose a “Flash size” option in the “Tools” menu to allocate memory for the File System (FS) before uploading the code to the microcontroller. The other options in the “Tools” menu can be kept as the default in the upload code process.

The microcontroller software is based on a web server, can handle file operations, and create a wireless access point network. The defined device network SSID (Service Set Identifier) is “photomer,” and no password is required to connect. This can be changed directly in the source code before uploading it to the board. The default fixed IP (Internet Protocol Address) defined by the ESP8266 Wi-Fi library is 192.168.4.1,^34^ and it was kept in the code. Although the ESP8266 Wi-Fi library’s maximum number of client connections is 8.^34^ To ensure the microcontroller runs smoothly, the code has been configured to allow only one client to connect to the device.

The client can access the web server by entering the end point URL (Uniform Resource Locator) specified in Table S1 into the address bar of any web browser on a PC or smartphone. In addition to the URL addresses, the table lists actions triggered by HTTP (Hypertext Transfer Protocol) requests and all possible HTTP responses returned by the web server.

The device can be used without a user interface, directly by the browser address bar. However, for a better user experience, an HTML user interface was created, which simulates a command-line interface (CLI), and the code lines are shown in Appendix A. Only two commands are answered by the CLI: “clear” and “out”. The first command deletes all the written lines in the CLI. The “out” command outputs the analog result given by the microcontroller.

To send the HTML files to the microcontroller board, a tool for uploading files, also written in HTML and JavaScript, was developed. To use it, the uploader HTML file must be opened in a web browser on a PC or smartphone connected to the 3D-photometer. The HTML file selected in the uploader program is sent to the microcontroller board via an HTTP POST request. Even the uploader itself can be sent and stored by the microcontroller board. To access the uploader tool stored on the microcontroller board, type the URL address http://192.168.4.1/pages/uploader.html into the web browser’s address bar, provided the tool is named uploader.html. All source code for the programs mentioned in this section can also be accessed in the git repository: https://github.com/kpi-jps/photometer_cli (Table S1).

The approximate total materials cost of the 3D-printed device was USD 25.85, excluding the country’s import taxes, and the cost of the 3D-printed filament, which was less than 100 g of ABS. Table S2 shows the materials cost of the electronic components used in the construction of the 3D-printed photometer.

### 2.2 Materials

Qualitative cellulose filter paper (Unifil) (80 g m^–2^) was used as a solid support. All reagents used were of analytical grade and were employed without prior purification. Urea (≥99%) was obtained from Synth; calcium chloride dihydrate (≥99%) was obtained from Neon; 4-dimethylamine cinnamaldehyde (*p*-DAC) (≥99.9%) was obtained from Sigma-Aldrich; and hydrochloric acid (≥99.9%) was obtained from Neon. The chromogenic solution consisted of a mixture of *p*-DAC at a concentration of 3.0% (w/v) in 0.50 mol L^–1^ hydrochloric acid with ethanol and water serving as solvents.

### 2.3 Samples

Different samples of ultra-high temperature (UHT) cow’s milk, purchased from a local market with varying expiration dates and batches, were used to evaluate the performance of the proposed analytical method. Milk samples were identified by their fat content as whole milk (3.0% fat) and skim milk (0.5% fat).

### 2.4 Salting out Procedure

The precipitation of proteins in milk (casein) was tested using different salts such as MgCl_2_·6H_2_O, NaCl, and CaCl_2_·2H_2_O at various concentrations (10, 20, and 30% w/v). The required amount of salt was added to 40 mL of milk (urea-free milk), and the solution was stirred for approximately 2 min to increase ionic strength and induce precipitation, followed by 400 μL of HCl (6.0 mol L^–1^) (100 μL/10 mL of milk), and stirred again for another 2 min. The solution was centrifuged at 9000 rpm for 9 min and filtered using a qualitative filter (80 g m^–2^) to separate the milk serum from the proteins and fat.

### 2.5 Sample Preparation Procedure

The clear filtrate was defined as milk serum and used to perform the derivatized reaction with the *p*-DAC reagent. Different volumes of the chromogen derivatizing reagent *p*-DAC (3.0% w/v in a solution of 0.5 mol L^–1^ HCl in ethanol) were tested after the salting out procedure described in Section 2.4. In a 10 mL volumetric flask, approximately 9.0 mL of milk serum was added together with 70 μL of *p*-DAC reagent, and the remaining volume was completed with milk serum. An orange-colored solution was formed in the presence of urea, whereas it was yellowish in its absence. The absorbance was measured using a UV–Vis spectrophotometer (UV-1800 Shimadzu) at 530 nm.

### 2.6 Thermogravimetric (TG) and Differential Thermal Analysis (DTA)

Characterization by TG and DTA was carried out on a TA Instruments SDT 2960 in at a flow rate of 10 °C min^–1^ using N_2_ as the carrier gas. The sample was heated in the temperature range of 30 to 600 °C. TG and DTA in solution were added to Al_2_O_3_ crucible pans with a volume of 90 μL.

### 2.7 Determination of Urea Using HPLC-UV–Vis

Urea quantification was performed by UV–Vis spectrophotometry using *p*-DAC as the derivatizing agent, yielding a colored compound with a maximum absorbance at 530 nm (Figure S2). Additionally, urea was quantified by a chromatographic method, with *p*-DAC derivatization performed on a Shimadzu LC-20AT HPLC coupled to a diode-array detector (UV–Vis) (SPD-M20A). The chromatographic conditions were Eclipse Plus C-18 reversed-phase column (100 mm × 4.60 mm), 5 μm Zorbax with a guard column (precolumn 4.0 mm × 3.0 mm × 50 mm), flow rate of 1.0 mL min^–1^, injection volume of 20 μL, and oven temperature of 30 °C. The linear gradient method was used for urea elution with a mobile phase composed of acetonitrile (ACN) and 0.1% formic acid (FA), varying ACN % from 10% to 70% in the first 7 min, then held at 70% until 9 min. After 9 min, the initial conditions for the subsequent analysis were reestablished, 10% ACN, with a total analysis time of 13 min.

All samples were prepared as described in Sections 2.4 and 2.5; however, for HPLC analysis, a 1:2 dilution of milk serum in water was performed, followed by derivatization with *p*-DAC. Subsequently, the solution was filtered through a 0.46 μm Nylon syringe filter to avoid precipitation into the column, and 20 μL of the filtrate was injected into the HPLC system. Urea quantification was determined from the relationship between the area of the chromatographic peak at a retention time of 3.82 ± 0.02 min, corresponding to the formation of the colored reaction product.

### 2.8 Mass Spectrometry Analysis of the Colored Reaction Product between Urea and *p*-DAC

The chromogenic reagent *p*-DAC can be used to determine urea by a colorimetric reaction in an acidic medium. The reaction involves condensing the urea’s protonated amino group with the carbonyl group present in its structure, forming an iminium salt (Figure 3).^25^ *p*-DAC is a yellow compound that turns orange in the presence of urea. To prove the proposed structure, a direct infusion-mass spectrometry analysis was performed using a SCIEX 3200 QTRAP mass spectrometer. The solutions of *p*-DAC + HCl and *p*-DAC + Urea + HCl, both diluted 100 times with acetonitrile/formic acid 99.9:0.1, were analyzed by mass spectrometry using an electrospray source (Turbo Ion Spray source) and direct infusion at 10 μL min^–1^. The ion source parameters used were: curtain gas at 10 psi, collision gas medium, ion spray voltage at 5500 V, source temperature at 200 °C, gas 1 and 2 at 13 psi, declustering potential at 70 V, entrance potential at 12 V, collision cell potential at 10 V (entrance) and 4 V (exit), and collision energy 25 V.

**Figure 3:**
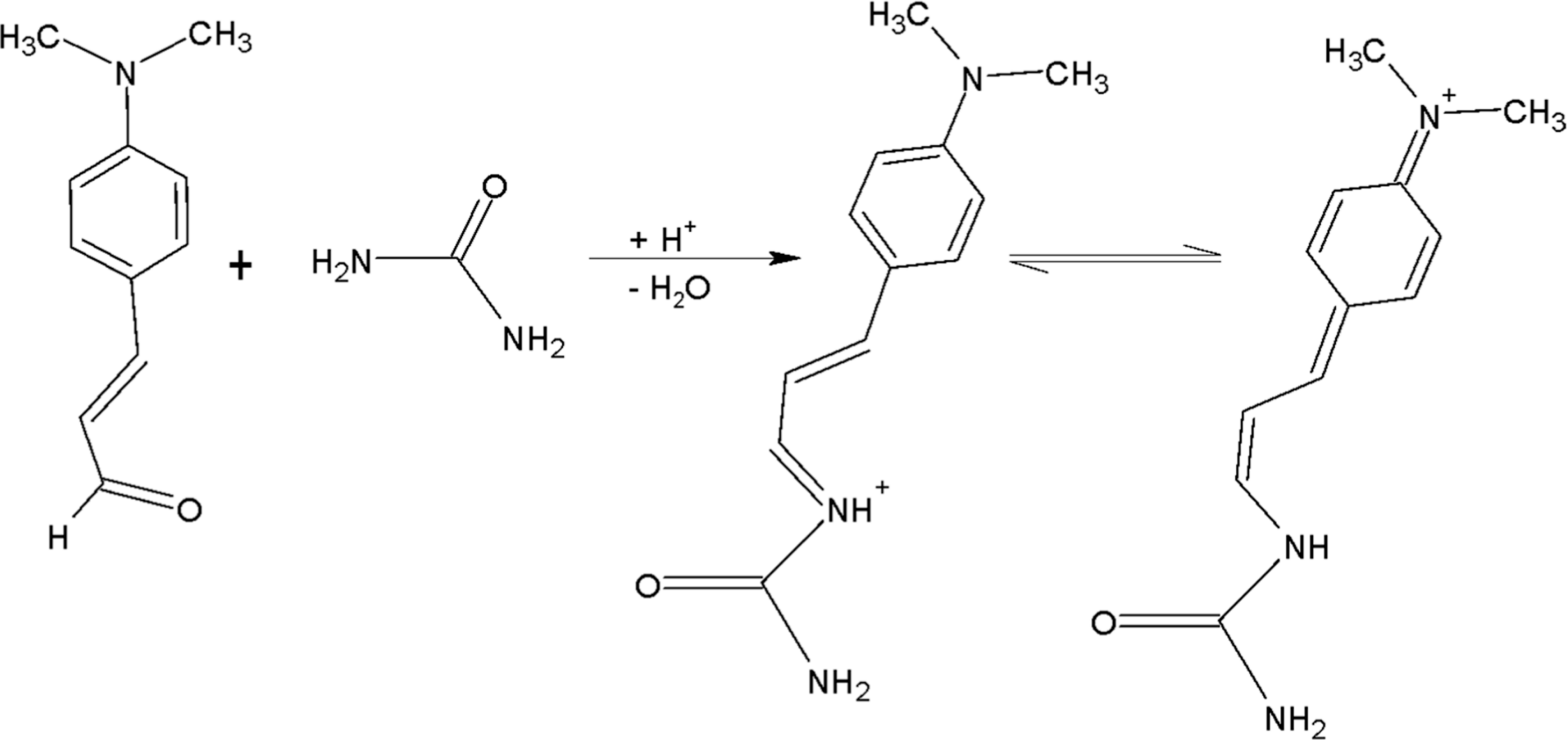
Reaction mechanism between urea and the chromogenic derivatizing reagent *p*-DAC.

### 2.9 Analytical Figures of Merit

The figure of merit validation method was conducted in accordance with the guidelines of regulatory agencies.^35,36^ The precision of the method was determined through repeatability, under the same operating conditions (equipment, analyst, and reagents). Selectivity was determined by comparing the sample quantification with that of a blank. The limits of detection (LOD) and quantification (LOQ) were determined from eqs 2 and 3, where SD is the standard deviation of the intercept, which was determined by evaluating the relationship between urea concentration in milk and absorbance at 530 nm in the spectrophotometer, 3D-printed photometer, and HPLC-UV–Vis.

2 $$LOD=\frac{3\times SD}{slope}$$

3 $$LOQ=\frac{10\times SD}{slope}$$

Additionally, linearity was evaluated by preparing solutions with urea concentrations ranging from 3.0 mg mL^–1^ to 40 mg mL^–1^ (0.05 mol L^–1^ to 0.67 mol L^–1^) and analyzing the relationship between concentration and analytical signal (absorbance or area), using at least six concentration points, each performed in triplicate. Recovery (Rec %) was determined in triplicate as a percentage from eq 4, using urea concentrations of 17 mg mL^–1^ (medium) and 28 mg mL^–1^ (high) from the calibration curve. The relative standard deviation (%RSD) values had to be less than 5% to be considered acceptable. Concentration_0_ refers to the urea concentration value obtained in milk samples spiked after the extraction procedure. Concentration_exp_ the urea concentration value obtained in milk samples spiked before the extraction procedure.

4 $$recovery\;\left(Rec\;\%\right)=\frac{{concentration}_{\exp}}{{concentration}_{0}}\times 100\%$$

## 3 Results and Discussion

### 3.1 Sample Preparation

#### 3.1.1 Salting-Out

To determine the optimal salt concentration for protein precipitation from UHT whole milk, the salting-out technique was used with MgCl_2_·6H_2_O, NaCl, and CaCl_2_·2H_2_O at 10, 20, and 30% w/v, following the procedures described in items 2.4 and 2.5. The results showed that, when using the MgCl_2_·6H_2_O salt at different concentrations, none were satisfactory, as complete precipitation was not achieved, resulting in a cloudy, whitish solution. Further, a yellow reaction product formed when derivatized with *p*-DAC, accompanied by precipitates that interfered with absorbance measurements, making the analysis difficult (Figure S3a). When using NaCl salt at concentrations of 10% and 20% w/v, the solution became cloudy without achieving complete precipitation. When 30% NaCl was used, the solution became a little clearer, but supersaturation was observed after precipitation with the appearance of NaCl crystals in the milk serum, which, after derivatization with *p*-DAC precipitated, forming a whitish-yellow solution that could lead to problems in the absorbance measurement (Figure S3b).

When 10–20% w/v CaCl_2_·2H_2_O was used, complete protein precipitation was not observed, resulting in a slightly cloudy solution that could make analysis difficult and contribute to experimental error. The best result was achieved with 30% CaCl_2_·2H_2_O, which yielded a clear solution with no supersaturation observed and maintained stability after derivatization with *p*-DAC. Furthermore, when 30% w/v CaCl_2_·2H_2_O was used with different types of UHT milk of varying fat composition, the same result was obtained, confirming that the first step of the sample preparation was reproducible (Figure S3c). All salt additions were compared to precipitation with 200 μL of HCl (6.0 mol L^–1^, 100 μL for each 10 mL milk) alone (without salting-out) added to 20.0 mL of milk (Figure S3a–c). Results show that acidification with HCl to pH 3.5–4.0 alone did not yield a clear solution, whereas the combination with salt concentration addition did. Figures S3a–c show that a slightly clear but whitish solution causes interference and problems with absorbance measurements.

Several studies have reported the influence of the destabilizing effect of the addition of salts on the coagulation, precipitation, and stability of casein (α, β, and κ) in milk samples, affected by pH, temperature, protein concentration, as well as salt concentration.^37,38^ These results are related to the decrease in repulsive electrostatic interactions between particles, which modifies the rheological properties of the suspension and alters its viscosity. This can lead to the formation of turbid suspensions and the precipitation of denser protein domains, thus facilitating micellization and aggregation.

Bauland et al.^39^ showed that the addition of the cation Mg^2+^ reduces electrostatic repulsion between caseins. At the same time, changes in pH and an increase in Ca^2+^ ionic concentration promote micelle aggregation. The primary role in the enzymatic coagulation and hydrolysis of κ-casein is attributed to the presence of colloidal Ca^2+^, which affects the kinetics of hydrolysis as well as the physicochemical properties of milk. While Na^+^ cations, due to their dehydrating effect, affect turbidity and emulsifying properties, requiring higher concentrations and a pH below 5.0 to reduce repulsive forces.^37,40,41^

Therefore, the addition of CaCl_2_ salts was expected to result in efficient precipitation of casein in milk at pH 4.0, promoting hydrolysis, interaction of electrostatic charges, structural variations, hydrophobicity, causing protein–protein changes, which consequently causes an increase in the size of protein micelles, generating micellization and aggregation, which leads to precipitation (Figure S3c).

### 3.2 TG and DTA of Urea in Aqueous Solution and Milk Serum

Hydrolysis significantly alters the thermal behavior of casein and urea, facilitating their decomposition at lower temperatures and modifying their thermal profiles as analyzed by thermogravimetric analysis (TG) and differential thermal analysis (DTA). The hydrolysis of milk casein by the addition of CaCl_2_·2H_2_O is observed through structural changes caused by the breakage of its peptide chains.^42,43^ In which the thermal degradation peaks can be associated with water loss, peptide degradation, and the burning of carbon residues. The DTA shows endothermic peaks corresponding to denaturation and exothermic peaks corresponding to decomposition. In the case of urea, decomposition produces ammonia and CO_2_, which affect the TG curve. The DTA exhibits endothermic peaks characteristic of the melting and thermal decomposition of urea.^44^

For solid urea, four distinct mass-loss stages were observed between 145 and 412 °C. The main decomposition occurred between 145 and 228 °C (68.7%), followed by minor losses up to 412 °C, corresponding to successive decomposition reactions of urea, HNCO, and NH_3_. The DTA analysis confirmed four endothermic events, including a melting peak at 136 °C and additional peaks related to thermal degradation^45^ (Figure S4a). CaCl_2_·2H_2_O showed two mass-loss stages: 1.2% loss up to 51.5 °C due to the release of surface water, and a 24% loss between 83.2 and 147.3 °C associated with dehydration, totaling 25.2% (Figure S4b). The DTA results showed two endothermic peaks consistent with these water losses.^46^

Urea in aqueous solution (20 mg mL^–1^) exhibited two decomposition stages: a major one between 29.5 and 74.5 °C (95.9% loss) and a smaller one between 123.1 and 178.5 °C (3.6%), resulting in almost complete mass loss (99.5%) (Figure S4c). The DTA analysis showed two endothermic peaks (73.9 and 176.2 °C), associated with the decomposition of urea into NH_3_, HCO_3_, and other products (H_2_O, CO_2_).^47^

In milk serum after precipitation with CaCl_2_·2H_2_O (30% w/v) and HCl (6.0 mol L^–1^), urea exhibited three decomposition stages with total mass loss of 74.3% (Figure S4d). The DTA showed two endothermic peaks (71.8 and 158.5 °C), associated with the thermal degradation of urea into NH_3_ and HCO_3_. The remaining losses were attributed to the decomposition of milk components, including fats, proteins, vitamins, salts, and lactose. Heating to 40 °C during the salting-out process caused only about 3.9% urea degradation, indicating minimal interference and confirming the method’s efficiency for protein precipitation.^48^

### 3.3 Kinetics and Stability of Urea with *p*-DAC

The optical stability of the reaction between urea and *p*-DAC was monitored over time by adding the chromogenic reagent immediately and measuring the absorbance at 530 nm every 0.5 min for up to 45 min. The colored reaction showed that the product formed exhibited a rapid increase in absorbance over the first 20 min, after which it remained stable for at least 45 min (Figure S5a). Therefore, a 30 min time was selected to achieve maximum absorbance and to provide good repeatability of the analyses.

To determine urea loss due to hydrolysis, the reaction was tested with different urea addition procedures at two concentrations: 8.0 mg mL^–1^ and 30 mg mL^–1^. The first is the joint addition of Salt + Urea_(s)_ (both solids). The second one, called Urea_(aq)_ + Salt, was prepared from a 500 mg mL^–1^ aqueous urea solution and added to the whole milk before salt precipitation. In the third procedure, Salt + Urea_(aq)_, an aqueous solution of urea at 500 mg mL^–1^ was added after precipitation, i.e., into the milk serum. The highest absorbance value (0.475 ± 0.009) was observed for the combined addition of salt and urea (both solids) at a concentration of 30 mg mL^–1^, followed by the addition of the aqueous urea solution after precipitation, which resulted in a 21.0% reduction in the signal (0.374 ± 0.020). The addition of the aqueous solution before precipitation led to a reduction of approximately 36.8% (0.300 ± 0.020) (Figure S5b). At a urea concentration of 8.0 mg mL^–1^, the signal remained more stable across the different procedures, with a slight difference between Salt + Urea_(s)_ (0.113 ± 0.007) and Salt + Urea_(aq)_ (0.096 ± 0.026), corresponding to a 15.0% reduction. The addition of aqueous urea before precipitation showed a greater absorbance reduction of 36.2% (0.072 ± 0.014).

As previously observed, the highest absorbance value was determined with the addition of urea and CaCl_2_·2H_2_O, both solids; therefore, the standard sample preparation procedure consisted of the addition of urea and CaCl_2_·2H_2_O, followed by 40 mL of milk and 400 μL HCl (6.0 mol L^–1^) to adjust the pH between 3.5 and 4.0. After shaking for approximately 2 min, the solution was centrifuged for 9 min at 9000 rpm, separated, and filtered through a Nylon filter. Next, approximately 9 mL of milk serum was transferred to a volumetric flask, and 70 μL of *p*-DAC (3.0% in ethanol/0.5 mol L^–1^ HCl) reagent was added, then more serum milk was added, completing the volume to 10 mL. After 30 min of reaction, the absorbance at 530 nm was measured. The entire sample preparation procedure is described in Figure 4.

**Figure 4:**
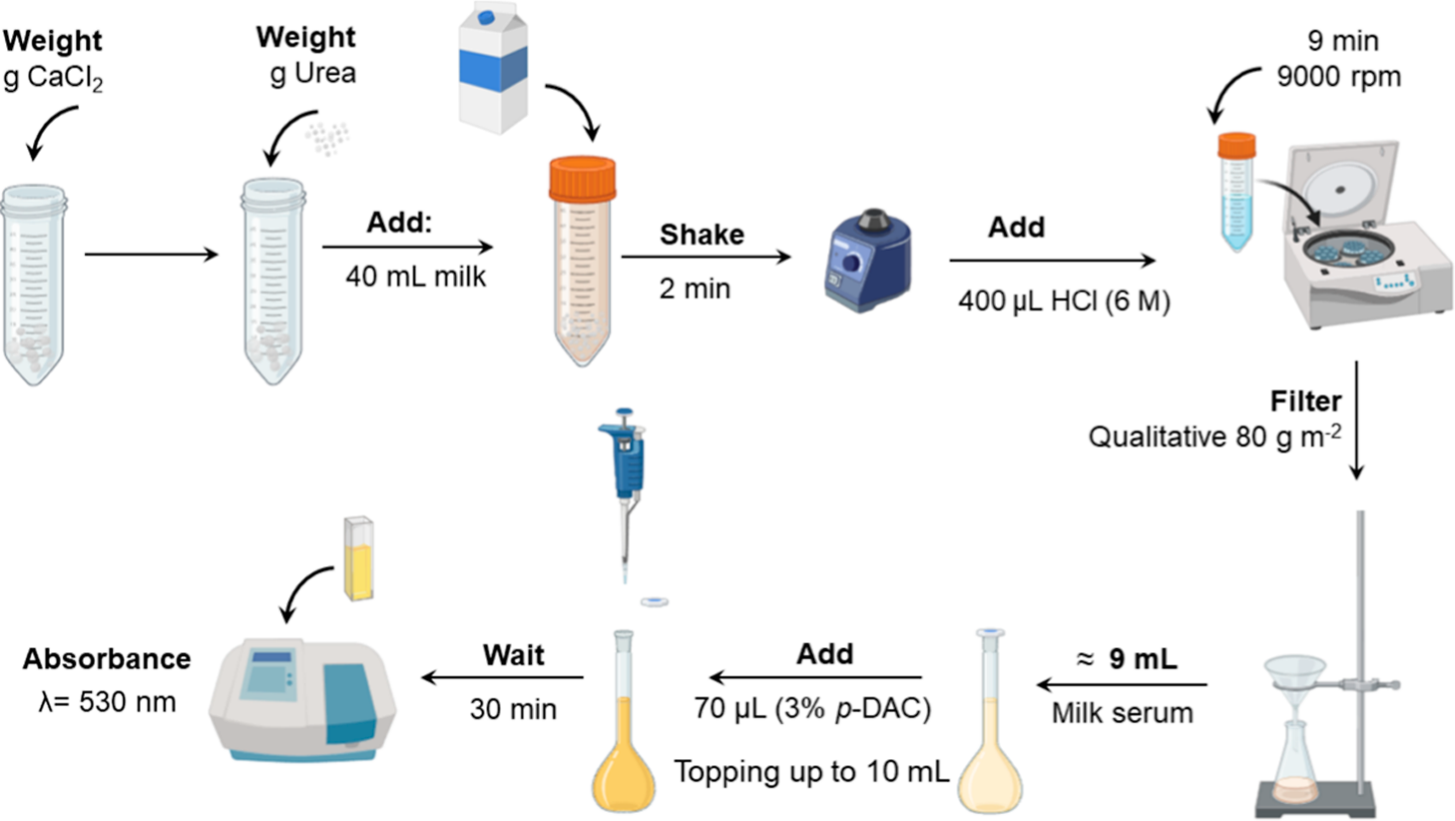
Sample preparation scheme for urea determination in milk using salting out with CaCl_2_·2H_2_O and colorimetric reaction with *p*-DAC.

### 3.4 Chemical Structure of the Urea–*p*-DAC Reaction Product by Mass Spectrometry

The colored reaction product between urea and *p*-DAC in an acidic medium was analyzed by mass spectrometry using direct infusion into an electrospray source. The chromogenic solution containing *p*-DAC and HCl was analyzed first, then reacted with urea and analyzed after 30 min. The colored solution presented a peak at *m*/*z* 61, demonstrating that not all the urea reacted with the chromogenic reagent, and another peak at *m*/*z* 176, indicating the excess of the *p*-DAC added to the solution (Figure S6a). The proposed reaction structure for the reaction, as suggested by Gigante,^25^ via the condensation of urea and *p*-DAC (Figure 3), was observed as an ion at *m*/*z* 218. Figure S6b illustrates the MS/MS spectrum of the precursor ion *m*/*z* 218, and Figure S7 shows the proposed fragmentation pathway for its collision-induced dissociation. Another possible product from the reaction of two molecules of *p*-DAC with one molecule of urea was not observed, indicating that the reaction follows the proposed condensation (Figure 3). To the best of our knowledge, this is the first work to elucidate the structure of the colored product formed between urea and *p*-DAC by mass spectrometry.

### 3.5 Analysis of the Photometric Device Performance

The experimental data obtained with the 3D-printed photometer decrease with increasing urea concentration, as indicated by a reduction in the electric current generated by the photodiode. The microcontroller (ESP32) has a 12-bit resolution, and it is capable of recording 4096 possible values between 0 and 3.3 V. When the blank reading is performed, the light that falls on the photodiode is maximum, generating a higher electric current that is converted into a higher voltage by the amplifier circuit (3300 mV). As the urea concentration in the solution increases, the microcontroller records lower voltage values. Thus, the lowest recorded voltage (800 mV) corresponds to the highest urea concentration. The determination of absorbance was calculated from eq 1.

Some limitations of the 3D-printed photometer are noted, including the use of a single LED and photodiode, which limit spectral selectivity and sensitivity compared with commercial instruments equipped with monochromators and advanced detectors. Furthermore, manual adjustment of LED intensity and detector gain can introduce operator-dependent variability.^49^ However, these limitations are offset by its low cost, low power consumption, ease of fabrication, maintenance, parts replacement, reliable urea quantification (see details in Section 3.6), and its favorable environmental profile, making the 3D-printed photometer a viable alternative for routine screening and rapid analysis rather than for high-precision measurements.

### 3.6 Analytical Figures of Merit for the Determination of Urea in Milk Samples

Technical Report No. 53 of the Ministry of Agriculture, Livestock, and Food Supply^50^ states that urea is not approved as a feed additive in Brazil. However, the Expert Committee on Feed Additives (JECFA) of the Food and Agriculture Organization (FAO) and the World Health Organization (WHO), in its 41st session 1993,^50,51^ permitted a maximum of 3.0% (w/v) (3.0 mg mL^–1^) without toxicological concerns. A key parameter indicating the nutritional quality of milk-producing animals is Milk Urea Nitrogen (MUN), which measures the amount of urea in milk and is typically expressed in mg dL^–1^. It is important to note that some of this urea may come from the nitrogen cycle (metabolic) of cows’ diets.

Coelho et al.^52^ reported ideal MUN values between 10 mg dL^–1^ and 18 mg dL^–1^ (0.1 mg mL^–1^–0.18 mg mL^–1^). Values outside this range indicate inadequate nutritional management, such as protein deficiency or excess in the diet, or adulteration. High MUN values above 60 mg dL^–1^ (0.6 mg mL^–1^) are related to lower protein content, which can compromise product yield and quality.^53^ In addition to MUN, Walstra and Jennes^54^ reported that milk contains a fraction of nonprotein nitrogen (NPN) compounds, including urea, creatine, and creatinine, which account for approximately 5% of the total nitrogen in the matrix. Therefore, in the present work, a working range of 3.0 to 40 mg mL^–1^ was used, with the minimum concentration being 5 times higher than that required to compromise the product’s quality, without posing any toxicological risk.

Analytical curves were obtained using the spectrophotometer and the 3D-printed photometer for whole milk (3.0% fat) and skim milk (0.5% fat) samples. The results showed good linearity *R* ^2^ between 0.986 and 0.997, a concentration range between 3.0 mg mL^–1^ and 40 mg mL^–1^, and a LOQ of 3.0 and 4.0 mg mL^–1^ for the spectrophotometer and the 3D-printed photometer, respectively. The 3D-printed photometer exhibited lower sensitivity (slope) than the curve obtained with a spectrophotometer (Figure 5a,b)^35^ for the reasons described in Section 3.5. However, it is worth noting that the 3D-printed photometer demonstrated suitable linearity, with results directly proportional to the analyte concentration in the sample, achieving precision values of less than 0.04% even in the presence of a complex matrix such as milk.

**Figure 5:**
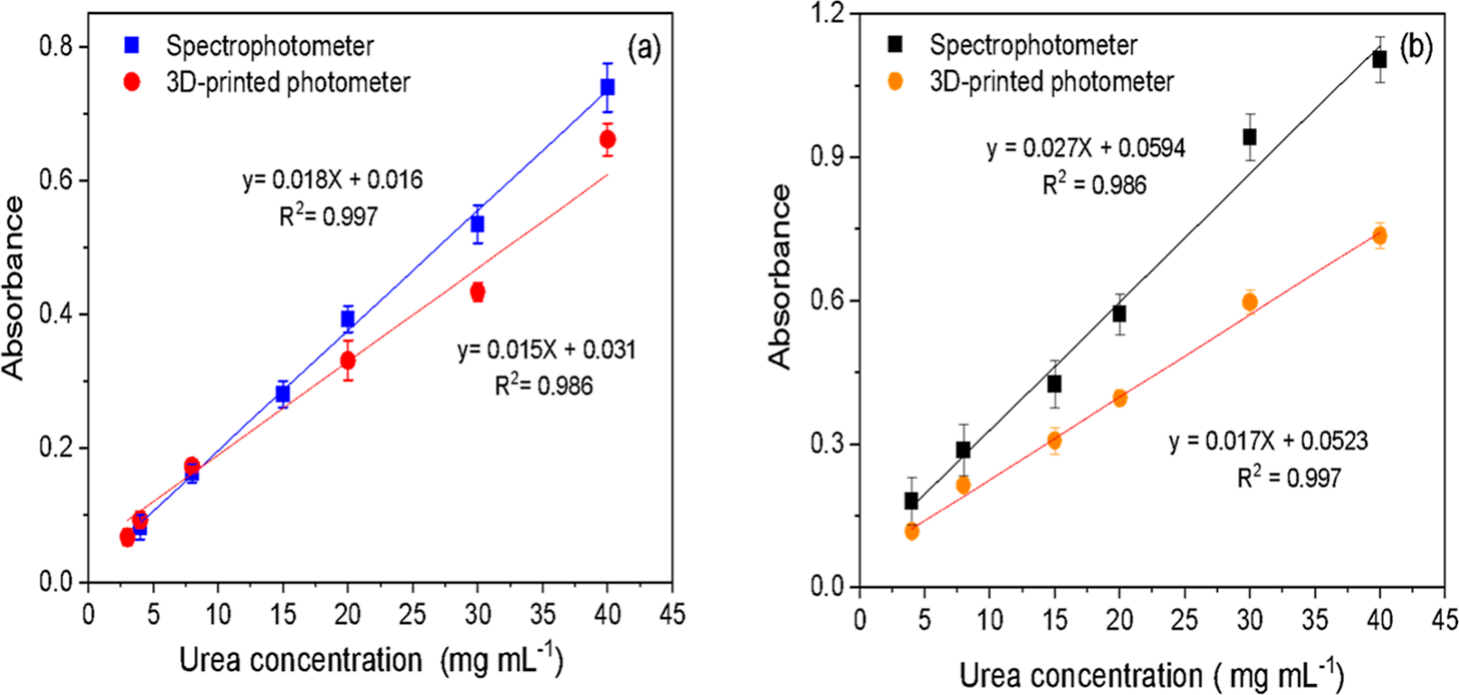
Analytical curves for urea quantification using a spectrophotometer and a 3D-printed photometer in (a) whole milk (3.0% fat), (b) skim milk (0.5% fat).

Skim milk (0.5% fat) showed a higher absorption value from the first concentration (3.0 mg mL^–1^), as well as an increased slope (sensitivity) of the curve, both for the spectrophotometer and for the 3D-printed photometer. Thus, the nutritional composition, especially total fat, influences the reaction between urea and *p*-DAC (Figure 5b).

The determination of figures of merit, which involves evaluating parameters that indicate the method’s reliability, was performed on samples of whole milk (3.0% fat) and skim milk (0.5% fat). The average Rec % at medium and high concentrations was 86.9–105%, with an RSD % (relative standard deviation) ranging from 0.01 to 5.38%. Values between 80 and 120% are generally acceptable for recovery tests as described in the AOAC guidelines for results validation.^55^ Intraday and interday precision were assessed at a concentration of 30 mg mL^–1^, and the results showed intraday precision values of 3.39 to 4.67%, demonstrating acceptable precision according to the test validation guidelines. The interday precision was compared across 3 days, ranging from 1.43% to 2.97%. The lower values reported, mainly by the 3D-printed photometer, demonstrate the repeatability of the proposed method and its suitability for determining urea in milk samples. All these results are summarized in Table 1.

**Table 1:** Comparison of figures of Merit for the 3D-Printed Photometer Device and a Spectrophotometer as a Reference Method for the Determination of Urea in Whole Milk UHT (3.0% Fat) and Skim Milk (0.5% Fat)

						Mean spike recovery ± % RSD			
Milk sample	Methods analytical	LOQ (mg mL^–1^)	Linear range (mg mL^–1^)	Calibration curve	*R* ^2^	Rec medium	Rec high	Intra-day precision (% RSD)	Inter-day precision (% RSD)
Whole milk (3.0% fat)	Spectrophotometer	3.0	3.0–40	*y* = 0.018 [Urea] + 0.016	0.997	101 ± 3.20	86.9 ± 2.01	4.45	1.43
	3D-printed photometer	4.0	4.0–40	*y* = 0.015 [Urea] + 0.031	0.986	105 ± 3.30	105 ± 0.01	3.79	1.47
Skim milk (0.5% fat)	Spectrophotometer	4.0	4.0–40	*y* = 0.027 [Urea] + 0.059	0.986	87.7 ± 3.51	103 ± 1.40	4.67	2.97
	3D-printed photometer	4.0	4.0–40	*y* = 0.017 [Urea] + 0.052	0.997	97.6 ± 5.38	101 ± 3.99	3.39	1.54

Other authors, such as Nandeshwar et al.,^27^ developed a sensor for quantifying urea in milk samples. The reaction was performed using the supernatant and a colorimetric reagent *p*-DMAB. The color intensity is proportional to the amount of chromogen produced. The optical detection system was a low-cost assembly of electronic components, including a blue LED, a photodiode, an electrical signal amplifier, a Bluetooth module, and a Raspberry Pi RP2020 microcontroller. Data was transmitted wirelessly to a smartphone for later analysis. The sensor demonstrated linearity across the three sample sets analyzed in triplicate, with *R* ^2^ = 0.720, over the concentration range of 0.1 to 0.7 mg mL^–1^. At low urea concentrations, this method showed limited linearity;^27^ the authors do not report its precision, but considerable deviations are observed at higher concentrations.

### 3.7 Urea Determination Using HPLC-UV–Vis

Urea quantification was also performed using HPLC-UV–Vis as a reference method to compare with the 3D-printed photometer in whole milk samples (3.0% fat). The results showed linearity with *R* ^2^ of 0.988, LOQ of 3.0 mg mL^–1^, and a working range between 3.0 and 40 mg mL^–1^ (Figure 6a). The chromatographic peak observed from the derivatization of urea with *p*-DAC is suitable, with a tailing factor (TF) of 1.5, with a peak height of 5%, in accordance with U.S. Pharmacopeia regulations,^56^ at the retention time (*R*_*t*_) of 3.82 ± 0.02 min, which showed proportional increase of the peak area with the increase in urea concentration (Figure S8). When the blank, as milk serum with *p*-DAC, was analyzed, no peak was observed at this retention time in the chromatogram (Figure 6b), demonstrating method selectivity.

**Figure 6:**
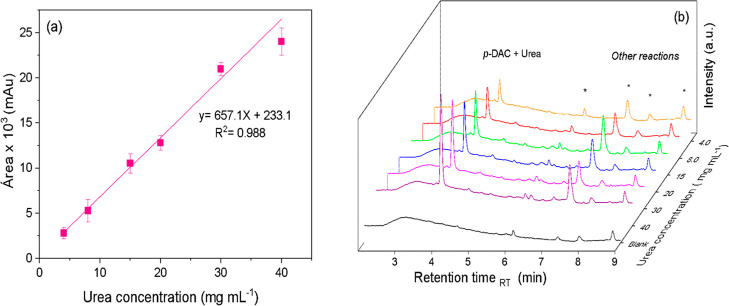
(a) Analytical curve for urea quantification using HPLC-UV–Vis in whole milk (3.0% fat), (b) chromatograms of the analytical curve of urea in whole milk. Peaks highlighted: derivatized reaction with *p*-DAC and *Other *p*-DAC reactions.

Similar to the spectrophotometer and the 3D-printed photometer, the figures of merit for the HPLC-UV–Vis were determined, with average and recovery values of 93.8% and 91.8%, respectively, and RSD % values of 5.65% and 5.20%, in accordance with validation guidelines.^35,36,57^ The intraday and interday precisions were low, at 0.96% and 1.43%, respectively (Table S3). These Rec % values were similar between those obtained with the spectrophotometer and the 3D-printed photometer (87% and 105%), and the RSD % was low, ranging from 0.01 to 3.30% (Tables 1 and S3). RSD values <5.0% indicate excellent repeatability of the method, as evidenced by strong agreement between measurements obtained with the spectrophotometer and the 3D-printed photometer for the same whole milk sample (3.0% fat). These results demonstrate that the method developed to determine urea with *p*-DAC in milk samples is precise and accurate, yielding consistent results despite operational variations (operator, equipment, day of analysis, batch, and milk sample).

### 3.8 Urea Determination Tests Using Different Milk Samples

Urea determination was performed on different milk samples, all of which contained the same urea concentration (30 mg mL^–1^). Quantification was conducted separately, accounting for each sample’s fat content. Whole milk, with a higher lipid content (3.0% fat), was quantified using analytical curves obtained from each of the equipment used (spectrophotometer, 3D-printed photometer, and HPLC-UV–Vis). In turn, the analytical curve obtained for skim milk (0.5% fat) was used to quantify semiskim milk (1.0% fat) and lactose-free milk (1.0% fat), which have similar lipid content (Tables 1 and S3).

The results showed that UHT whole milk with 3.0% fat was the one that best adjusted to the proposed method for both the 3D-printed photometer and the standard equipment, such as the spectrophotometer and HPLC-UV–Vis, with concentrations ranging from 28.0 to 30.0 mg mL^–1^ and %RSD between 1.70 and 2.47%. For skim (0.5% fat), semiskim (1.0% fat), and lactose-free (1.0% fat) milk samples, it was observed that the 3D-printed photometer showed concentrations between 23.3 and 28.0 mg mL^–1^ with %RSD 2.30 to 4.60, compared with 24.9–28.9 mg mL^–1^ of the spectrophotometer with %RSD 1.70 to 3.29. The HPLC-UV–Vis results ranged from 27.9 to 30.8 mg mL^–1^, with %RSD values of 0.11 to 2.47 (Table 2). The 3D-printed photometer showed lower sensitivity and precision in milk samples with lower lipid content (skim, semiskim, and zero lactose), as well as greater variation in the results (≤4.60%) (Table 2), compared to the spectrophotometer (≤3.29%) and HPLC-UV–Vis (≤2.47%). However, these %RSD remain below 5.00%, indicating that the results were consistent, repeatable, and acceptable based on the criteria defined in analytical method guidelines.^35,36,57^ The 3D-printed photometer proved to be accurate, reproducible, portable, and low-cost, achieving precision comparable to that of standard methods for determining urea in milk samples, thereby yielding precise results.

**Table 2:** Quantification of Urea (30 mg mL^–1^) in Different Milk Samples Using a 3D-Printed Photometer, Spectrophotometer, and HPLC-UV–Vis

	30 mg mL^–1^ of urea + % RSD		
Milk samples UHT (% fat)	3D-printed photometer	Spectrophotometer	HPLC-UV–Vis
Whole (3.0%)	28.0 ± 2.30	29.0 ± 1.70	30.0 ± 2.47
Skim (0.5%)	23.3 ± 4.00	28.9 ± 2.20	27.9 ± 1.70
Semiskim (1.0%)	23.3 ± 4.60	25.0 ± 3.29	30.8 ± 2.10
Lactose-free (1.0%)	24.4 ± 2.65	24.9 ± 2.51	29.8 ± 0.11

### 3.9 Statistical Analysis: ANOVA, *t*-Test, and *F*-Test, Comparison between 3D-Printed Photometer, Spectrophotometer, and HPLC-UV–Vis

The results of the ANOVA analysis showed that the analytical curves for the different milk samples are adjusted, since the tabulated *F* values are greater than the significance values *F*_tab_ > *F*_meaning_ (Table S4), thus, the adjusted mathematical models describe well the analyte concentration obtained in each equipment. In addition, the results obtained from the three different equipment were compared in pairs to determine whether there was variance in the means of the analytical values, using the *t*-test and *F*-test at 95% confidence.

The results of Rec % medium and high (17 and 28 mg mL^–1^) (Tables 1 and S3), were used for hypothesis testing and significance values, based on the null hypothesis (distribution between the two groups is not different) and alternative hypotheses (there is a difference between the groups or association between the variables) *F*-test and *t*-test among whole and skim milk samples, as well as among the equipment used for urea quantification (3D-printed photometer, spectrophotometer, and HPLC-UV–Vis).

The *t*-test_(95%)_ for whole milk samples (3.0% fat) for the comparison between the 3D-printed photometer and spectrophotometer yielded a two-tailed *p*-value of 0.81, which is greater than the predetermined *p*-value = 0.05. The *F*-test for significance comparison resulted in a one-tailed *p*-value of 0.17, greater than α = 0.05, thus confirming that the variances are equivalent. The unpaired *t*-test for the means of the same values, assuming equivalent variances, resulted in a two-tailed *p*-value equal to 0.86, greater than α = 0.05. Based on these statistical tests, the null hypothesis is supported, with the *p*-value indicating there is no statistical difference between these two detection methods when quantifying urea concentration (Table S4).

The statistical comparison for the results obtained with the 3D-printed photometer and HPLC-UV–Vis was performed similarly using whole milk samples; the *t*-test_(95%)_ was observed for the pairs of means of the six concentrations, with the same confidence level, resulting in a two-tailed *p*-value of 0.63 greater than α = 0.05, showing equivalent variances. The *F*-test for analysis of variances was performed. The resulting one-tailed *p*-value was 0.39, greater than α = 0.05, so the variances are equivalent (Table S4). Finally, the unpaired *t*-test, assuming equal variances, resulted in a two-tailed *p*-value of 0.79, which is greater than α = 0.05. Therefore, there is no significant difference between the 3D-printed photometer and HPLC-UV–Vis in measuring urea concentration.

In the statistical comparison of skim milk samples between the 3D printed photometer and the spectrophotometer, the *t*-test_(95%)_ obtained a two-tailed *p*-value of 0.97, greater than 0.05. The *F*-test showed a one-tailed *p*-value of 0.42, which is greater than α = 0.05, thus confirming that the variances are equivalent between the photometer and the spectrophotometer for urea quantification in skim milk samples (Table S4).

Based on the results from ANOVA, *t* tests, and *F*-tests, it was possible to verify that the 3D-printed photometer shows accuracy comparable to that of robust equipment, such as the spectrophotometer and the HPLC-UV–Vis, with no significant differences in the urea values obtained.

### 3.10 Comparison of the 3D-Printed Photometer Performance

Table 3 compares three 3D-printed photometers, including characteristics such as electronic components, microcontrollers, LEDs, material cost, and the reaction in which they were tested. González-Laprea et al.^58^ developed a 3D-printed photometer with an RGB LED (460–620 nm), with an Arduino-Python interface. The results showed an adjusted *R* ^2^ of 0.999 for the three colorimetric reactions tested: Cr­(NO_3_)_3_, KMnO_4_, and CuSO_4_·5H_2_O. The photometer cost USD104, which was relatively high compared to other models. It proved to be efficient, portable, and easy to maintain. However, the use of RGB LEDs limits the applicability of this device, since in reactions with Cr­(NO_3_)_3_ and CuSO_4_·5H_2_O (λ_max_ = 400 nm and λ_max_ = 670 nm, respectively), the RGB LEDs only absorb 30% of the irradiation. Based on the AGREE metric system (0–1), which evaluates the greenness (sustainability) of analytical methods according to the 12 principles of Green Analytical Chemistry (GAC),^59^ this study achieved a score of 0.51, which is classified as moderately green, primarily due to the use of oxidizing solvents, waste generation, and limited use of renewable sources.

**Table 3:** Comparison of 3D-Printed Photometers Found in the Literature with This Work

Device	Analyte	LED/nm	Price/USD	Electronic Components	Reaction	Reagents	LOQ	AGREE	Ref
3D-Printable RGB LED photometer	KMnO_4_	520	104	Light sensor BH1750 Arduino Nano	Complex in aqueous solution	KMnO_4_	0.10 × 10^–3^ mol L^–3^	0.51	^58^
Portable LED-based photometer	Urea in milk	517	35	TSL257	Berthelot reaction	Urea, acetic acid, salicylate, nitroprusside, and buffer	5.00 × 10^–2^ mg mL^–1^	0.57	^19^
3D-printed photometer	Natural raw pigments *Spirulina* sp	633	Not found	TSL250, IC-TSL250, Arduino Uno	Natural pigment crude extract	CaCO_3_, ascorbate, methanol	3.44% v/v	0.64	^60^
3D-printed photometric	Urea in milk	520	26	BPW34 OPA344PA Arduino ESP8866	Urea and *p*-DAC	Urea, CaCl_2_, HCl, *p*-DAC	4.00 mg mL^–1^	0.77	This work

Suarez et al.^19^ developed a portable LED-based photometer. The authors did not provide detailed technical information about the photometer, except for the LEDs’ wavelength range (639–517 nm), photodiode specifications, and the optical path. The cost was USD 35, and it was used to determine urea in milk using the Berthelot reaction. The results showed high efficiency, with good Rec % > 95% and a LOQ of 0.05 mg mL^–1^. The method required multiple steps (seven) and the use of reagents such as acetic acid, sodium salicylate, sodium nitroprusside, and phosphate buffer. The urea quantification was based on measurements of the sample potential difference (mV), the potential difference in the dark for background correction, and the potential difference of water as a blank, thus requiring additional measurements using a multimeter. The AGREE score was 0.57, classifying this technique as moderately green and environmentally acceptable, and indicative of a well-designed analytical method. The score reflects factors such as the use of toxic reagents, the required sample volume, and the number of procedural steps in the analytical methodology.

Kurniawan and Adhiwibawa^60^ developed a simple 3D-printed photometer for the determination of natural raw pigments in crude extract. The photometer consists of a 633 nm LED, a TSL250 photodiode, a resistor, and an Arduino Uno microcontroller. Detailed technical information about the equipment was presented, demonstrating the photometer’s simplicity and reproducibility. The results showed *R* ^2^ = 0.967 and LOQ 3.44% (v/v). The photometer proved less accurate than the spectrophotometer due to sample dilution and an inaccurate adjustment of the initial LED intensity using the variable resistor. However, it demonstrated a response similar (linear relationship) to that of a commercial instrument (spectrophotometer). In the AGREE metric analysis, the analytical method is moderately green, with a value of 0.64, indicating good efficiency, low energy consumption, reduced waste generation, and adequate safety for the operator and the environment.

This study demonstrates the potential of a 3D-printed photometer for quantifying urea in milk samples, highlighting its sustainability through the use of ABS filament, ease of access to materials and parts, maintenance, and scalability of its electronic components. The USB power supply provides a regulated voltage, reducing the risk of damage from overload. Additionally, wireless communication enables real-time data acquisition, monitoring of complete production lines, and operation in remote locations. The device is readily adaptable to different colorimetric reactions, requiring only the replacement of the LED with one matching the appropriate wavelength for the target analyte and reaction. Manual control of the LED intensity, combined with adjustable detector gain, prevents signal saturation and optimizes sensitivity, allowing operation under ideal analytical conditions. Due to its low cost and simple design, the system is well-suited for scalable manufacturing, thus reducing the cost per unit. No interference was observed in the optical measurements, which can be attributed to the precise fit of the device’s components, which prevent external light from entering and ensure accurate signal acquisition. Furthermore, the implemented noise-reduction strategies, including moving-average per-measurement processing and a low-pass filter, in the electronic circuit proved efficient, demonstrating the device’s potential for applications in analytical chemistry. The method demonstrated strong environmental sustainability performance, characterized by a low ecological impact, efficient resource use, and minimal waste generation. Accordingly, it achieved an AGREE score of 0.77, which aligns with most GAC principles and is considered a sustainable method with excellent ecological performance.

## 4 Conclusions

The derivatization reaction using *p*-DAC for the determination of urea in milk samples performed with a 3D-printed photometer, proved to be stable, yielding accurate, precise, and reliable results comparable to those obtained by spectrophotometry and high-performance liquid chromatography, across different types of milk. As well as the method developed for sample preparation using the salting-out protocol with CaCl_2_·2H_2_O salt in an acidic medium, which was essential to remove interferents and sample compounds to obtain a clear solution for a successful derivatization with *p*-DAC.

The methods for quantifying urea in milk did not show any lack of adjustment during method validation, in both the designed device and the standard equipment, which produced analytical calibration curves with similar linear working ranges (4.0 mg mL^–1^–40 mg mL^–1^) and LOQs relevant to milk safety analysis. The recovery and repeatability values were in accordance with official validation guidelines. Statistical tests enable us to demonstrate that the results obtained with the 3D-printed photometer are similar to those obtained by spectrophotometry or high-performance liquid chromatography, even though the analysis matrix is complex.

From a technological perspective, the modular architecture of the 3D-printed photometer enables future adaptations to different colorimetric reactions by replacing LEDs, as well as integration with wireless systems, digital platforms, and automated production lines. These features demonstrate the potential of the developed device to expand access to analytical technologies, including quality control in the dairy industry, routine field analyses, and educational applications due to its portability, operational simplicity, and low material cost (≈USD 26), making it a sustainable and scalable solution.

## Supplementary Material



## Data Availability

Data used is available throughout the manuscript text. The data and findings obtained in this study are available upon request from the corresponding author. P.C.F.L.G.

## Notes

Spectra (green LED, UV urea + *p*-DAC), web service sketch (data collection, end point and URL address), estimated cost, photos (milk protein precipitation and colorimetric reaction), TG/DTA analyses, kinetics of the urea + *p*-DAC reaction, urea addition procedures, spectra (full scan, MS/MS fragmentation and fragmentation pathway for ion *m*/*z* 218), figures of merit by HPLC-UV–Vis and statistical analysis test (ANOVA, *t*-test and *F*-test) in Figures S1–S8 and Tables S1–S4 (PDF)
