# Supplementary material for: Development of a 3D-Printed Photometric Device for the Determination of Urea in Milk Samples
Source: ACS Omega. 2026 May 21;11(21):30403–16. doi: 10.1021/acsomega.5c09412 (PMC13234886; doi:10.1021/acsomega.5c09412)
Supplement: Supplementary file 1 [file ao5c09412_si_001.pdf]

Development of a 3D-printed photometric device for the determination of urea in milk samples

Natália dos Santos Trindade[1], Saidy Cristina Ayala-Durán[1], Gabriel Baroffaldi Piassalonga[1], João Pedro Silva[1], Eduardo Luiz Rossini[1], Maria Valnice Boldrin Zanoni[1], Paulo Clairmont Feitosa de Lima Gomes[1*].

[1]Sao Paulo State University (UNESP), Institute of Chemistry, Department of Analytical Chemistry, Physical Chemistry and Inorganic Chemistry, National Institute for Alternative Technologies of Detection, Toxicological Evaluation and Removal of Micropollutants and Radioactives (INCT-DATREM), São Paulo State University (UNESP), Araraquara, SP, 14800-060, Brazil.

***Corresponding author:** Paulo Clairmont Feitosa de Lima Gomes

Address: Rua Prof. Francisco Degni 55, Araraquara, SP, 14800-060, Brazil

Phone: +55 (16) 3373 9613

E-mail: paulo.clairmont@unesp.br

Figure S1- Emission spectrum of Green LED (high brightness) measured by a spectrometer Red Tide USB 650 (Ocean Optics).

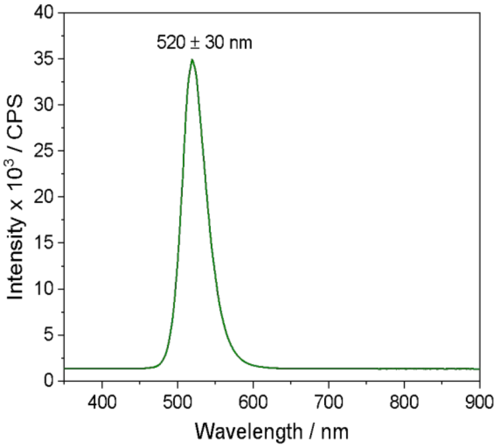

Appendix A- Web Service sketch for collecting data from the 3D-printed photometer.

```
#include <ESP8266WiFi.h>
#include <FS.h>
#include <LittleFS.h>

const String SSID = "photometer_cli";
const String pagesPath = "/pages/";
bool connected = false;
const int readingNumbers = 10;
String ip;

// Set web server port number to 80
WiFiServer server(80);


void hasClientConnectedToWiFi() {
  if (WiFi.softAPgetStationNum() > 0) {
    connected = true;
    return;
  }
  connected = false;
}

String extractFromString(String str, String startChars, String endChars) {
  int i = str.indexOf(startChars);
  int j = str.indexOf(endChars);
  String result = str.substring(i + startChars.length(), j);
  return result;
}

int reading() {
  int i = 0;
  int sum = 0;
  while (i < readingNumbers) {
    sum += analogRead(A0) * (3300 / 1024);
    i++;
    delay(1);
  }
  return sum / readingNumbers;
}

// for basic responses
void okResponse(WiFiClient client) {
  String content = "{\"response\" : \"OK\" }";
  String responseHeaders = "HTTP/1.1 200 OK\n";
  responseHeaders += "Access-Control-Allow-Origin: *\n";
  responseHeaders += "Connection: Keep-Alive\n";
  responseHeaders += "Keep-Alive: timeout=10, max=200\n";
  responseHeaders += "Content-type: application/json \n";
  responseHeaders += "Content-Length:" + String(content.length()) + "\n";
```

```
  responseHeaders += "\n";
  Serial.println(responseHeaders + "\n");
  client.print(responseHeaders);
  client.print(content);
}

// for files
void okResponse(WiFiClient client, String filePath, String contentType) {
  File file = LittleFS.open(filePath, "r");
  String responseHeaders = "HTTP/1.1 200 OK\n";
  responseHeaders += "Access-Control-Allow-Origin: *\n";
  responseHeaders += "Connection: Keep-Alive\n";
  responseHeaders += "Keep-Alive: timeout=10, max=200\n";
  responseHeaders += "Content-type: " + contentType + " \n";
  responseHeaders += "Content-Length:" + String(file.size()) + "\n";
  responseHeaders += "\n";
  Serial.println(responseHeaders);
  client.print(responseHeaders);
  long count = 0;
  while (count < file.size()) {
    char c = file.read();
    client.print(c);
    count++;
  }
  file.close();
}

// for JSON
void okResponse(WiFiClient client, String content) {
  String responseHeaders = "HTTP/1.1 200 OK\n";
  responseHeaders += "Access-Control-Allow-Origin: *\n";
  responseHeaders += "Connection: Keep-Alive\n";
  responseHeaders += "Keep-Alive: timeout=10, max=200\n";
  responseHeaders += "Content-type: application/json \n";
  responseHeaders += "Content-Length:" + String(content.length()) + "\n";
  responseHeaders += "\n";
  Serial.println(responseHeaders + "\n");
  client.print(responseHeaders);
  client.print(content);
}

void badRequest(WiFiClient client) {
  String content = "{\"response\" : \"Bad Request\"}";
  String responseHeaders = "HTTP/1.1 400 Bad Request \n";
  responseHeaders += "Access-Control-Allow-Origin: *\n";
  responseHeaders += "Content-type: application/json \n";
  responseHeaders += "Content-Length:" + String(content.length()) + "\n";
  responseHeaders += "\n";
  Serial.println(responseHeaders + "\n");
  client.print(responseHeaders + content);
}

void forbidden(WiFiClient client) {
  String content = "{\"response\" : \" Forbidden\"}";
  String responseHeaders = "HTTP/1.1 403 Forbidden \n";
  responseHeaders += "Access-Control-Allow-Origin: *\n";
```

```
  responseHeaders += "Content-type: application/json \n";
  responseHeaders += "Content-Length:" + String(content.length()) + "\n";
  responseHeaders += "\n";
  Serial.println(responseHeaders + "\n");
  client.print(responseHeaders + content);
}

void notFoundResponse(WiFiClient client) {
  String content = "{\"response\" : \"Not Found\"}";
  String responseHeaders = "HTTP/1.1 404 Not Found \n";
  responseHeaders += "Access-Control-Allow-Origin: *\n";
  responseHeaders += "Content-type: application/json \n";
  responseHeaders += "Content-Length:" + String(content.length()) + "\n";
  responseHeaders += "\n";
  Serial.println(responseHeaders + "\n");
  client.print(responseHeaders + content);
}

void preFlightResponse(WiFiClient client) {
  String responseHeaders = "HTTP/1.1 204 No Content\n";
  responseHeaders += "Connection: Keep-Alive\n";
  responseHeaders += "Keep-Alive: timeout=10, max=200\n";
  responseHeaders += "Access-Control-Allow-Origin: *\n";
  responseHeaders += "Access-Control-Allow-Methods: POST, GET\n";
  responseHeaders += "Access-Control-Allow-Headers: access-control-allow-origin,content-disposition,content-
type\n";
  responseHeaders += "\n";
  Serial.println(responseHeaders + "\n");
  client.print(responseHeaders);
}

long uploadFile(WiFiClient client, String fileName) {
  String path = pagesPath + fileName;
  long bytes = 0;
  if (LittleFS.exists(path))
    LittleFS.remove(path);
  File file = LittleFS.open(path, "w");
  while (client.available()) {
    char c = client.read();
    file.print(c);
    bytes++;
  }
  file.close();
  return bytes;
}

void handleClient(WiFiClient client, String requestHeaders) {
  //preflight
  if (requestHeaders.indexOf("OPTIONS /upload/") != -1) {
    preFlightResponse(client);
    return;
  }
  // upload pages
  if (requestHeaders.indexOf("POST /upload/") != -1) {
    if (requestHeaders.indexOf("text/html") == -1 || requestHeaders.indexOf("filename=") == -1) {
      badRequest(client);
```

```
      return;
    }
    String name = extractFromString(requestHeaders, "/upload/", " HTTP");
    long bytes = uploadFile(client, name);
    String content = "{\"bytes\" : " + String(bytes) + "}";
    okResponse(client, content);
    return;
  }
  // delivery html index page
  if (requestHeaders.indexOf("GET / ") != -1) {
    String path = pagesPath + "index.html";
    if (LittleFS.exists(path)) {
      okResponse(client, path, "text/html");
      return;
    }
  }
  // delivery the others page
  if (requestHeaders.indexOf("GET /pages/") != -1) {
    Serial.println("pages");
    String name = extractFromString(requestHeaders, "/pages/", " HTTP");
    String path = pagesPath + name;
    Serial.println(path);
    if (LittleFS.exists(path)) {
      okResponse(client, path, "text/html");
      return;
    }
  }
  // list all pages
  if (requestHeaders.indexOf("GET /list/pages ") != -1) {
    String content = "[";
    File root = LittleFS.open(pagesPath, "r");
    if (root && root.isDirectory()) {
      File file = root.openNextFile();
      if (file) {
        content += "\"";
        content += String(file.name());
        content += "\"";
        file = root.openNextFile();
        while (file) {
          content += ", \"";
          content += String(file.name());
          content += "\"";
          file = root.openNextFile();
        }
      }
    }
    content += "]";
    okResponse(client, content);
    return;
  }
  // clear all pages
  if (requestHeaders.indexOf("GET /clear/pages ") != -1) {
    File root = LittleFS.open(pagesPath, "r");
    if (root && root.isDirectory()) {
      File file = root.openNextFile();
      while (file) {
```

```
        String name = String(file.name());
        String path = pagesPath + name;
        LittleFS.remove(path);
        file = root.openNextFile();
      }
    }
    okResponse(client);
    return;
  }

  //get output
  if (requestHeaders.indexOf("GET /out") != -1) {
    String output = String(reading());
    String content = "{\"millivots\":" + output + "}";
    okResponse(client, content);
    return;
  }

  notFoundResponse(client);
}

void setup() {
  Serial.begin(115200);
  if (!LittleFS.begin()) {
    Serial.println("LittleFS Mount Failed");
    while (true)
      ;
  }
  WiFi.setSleepMode(WIFI_NONE_SLEEP);
  if (!WiFi.softAP(SSID,"", 1, 0, 1)) {
    Serial.println("Webserver failed starting...");
    return;
  }
  ip = WiFi.softAPIP().toString();
  Serial.print("Soft-AP IP address = ");
  Serial.println(ip);  // standard ip = 192.168.4.1
  Serial.println("Webserver started...");
  server.begin();
}

void loop() {
  hasClientConnectedToWiFi();
  WiFiClient client = server.available();
  if (client) {
    String headers = "";
    Serial.println("new client connected...");
    // an http request header ends with a blank line
    bool currentLineIsBlank = true;
    while (client.connected()) {
      if (client.available()) {
        char c = client.read();
        headers += c;
        Serial.print(c);
        // if you've gotten to the end of the line (received a newline
        // character) and the line is blank, the http request header has ended,
        // so you can send a reply
```

```
        if (c == '\n' && currentLineIsBlank) {
          handleClient(client, headers);
          break;
        }
        if (c == '\n') {
          currentLineIsBlank = true;
        } else if (c != '\r') {
          currentLineIsBlank = false;
        }
      }
    }
    // give time to the browser
    delay(500);
    // close the connection:
    client.stop();
    Serial.println("client disconnected...");
  }
  delay(100);
}
```

Table S1- 3D-photometer Web server endpoint URL address, actions, and possible HTTP responses.

Endpoint URL addresses	Action	Responses
http://192.168.4.1/	Delivery an HTML file named index.html if it is stored on the microcontroller board	HTTP response of status code 200 (OK status code) with an index.html file in the response body<br><br>HTTP response of status code 404 (Not Found status code) if not exist the index.html
http://192.168.4.1/pages/<filename>.html *	Deliver a required HTML file if it is stored by the microcontroller board	HTTP response of status code 200 with the required HTML file in the response body<br><br>HTTP response of status code 404 if not exist the required HTML file
http://192.168.4.1/list/pages	List all HTML files stored by the microcontroller board	HTTP response of status code 200 with a response body containing a JSON JavaScript array representation, empty or filled with names of all HTML files
http://192.168.4.1/clear/pages	Delete all HTML files stored by the microcontroller board	HTTP response of status code 200, even removing or not html files
http://192.168.4.1/out	Output the analog result in millivolts	HTTP response of status code 200 with a response body containing a JSON structure representing a JavaScript object with the parameter "millivolts" and your numeric value. Ex: {"millivolts": 200}

*<filename> represents the name of html file uploaded and saved in the microcontroller board's file system.
**URL addresses different from those presented in the table will give responses with a status code 404.

Table S2- The estimated approximate cost of the 3D-printed photometer.

Electronic components	Price (USD)*
BPW 34	2.34
OPA344PA	1.27
Green LED Diode Lights (5 mm)	0.38
ESP 8266 D1 mini	2.60
1 MΩ potentiometer	8.99
PCB - FR-4 Standard TG 135-140, 1.6 mm board width, HASL surface	2.20
Resistors	2.00
USB-C	6.07
**Total price estimated**	**25.85**

Price estimated on https://www.amazon.com/. Date: 19/05/2025.

Figure S2- UV-Vis spectra of urea and the product from the reaction between urea and *p*-DAC 3.0% w/v (0.5 mol L^{-1} HCl/ ethanol)

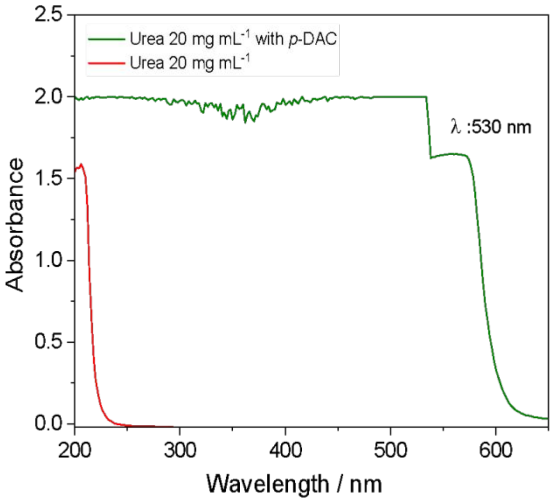

Figure S3- (a) Photos of the precipitation of whole milk proteins using salts **(a)** $MgCl_26H_2O$; **(b)** NaCl; **(c)** $CaCl_22H_2O$ in concentrations of 10, 20, and 30 %w/v.

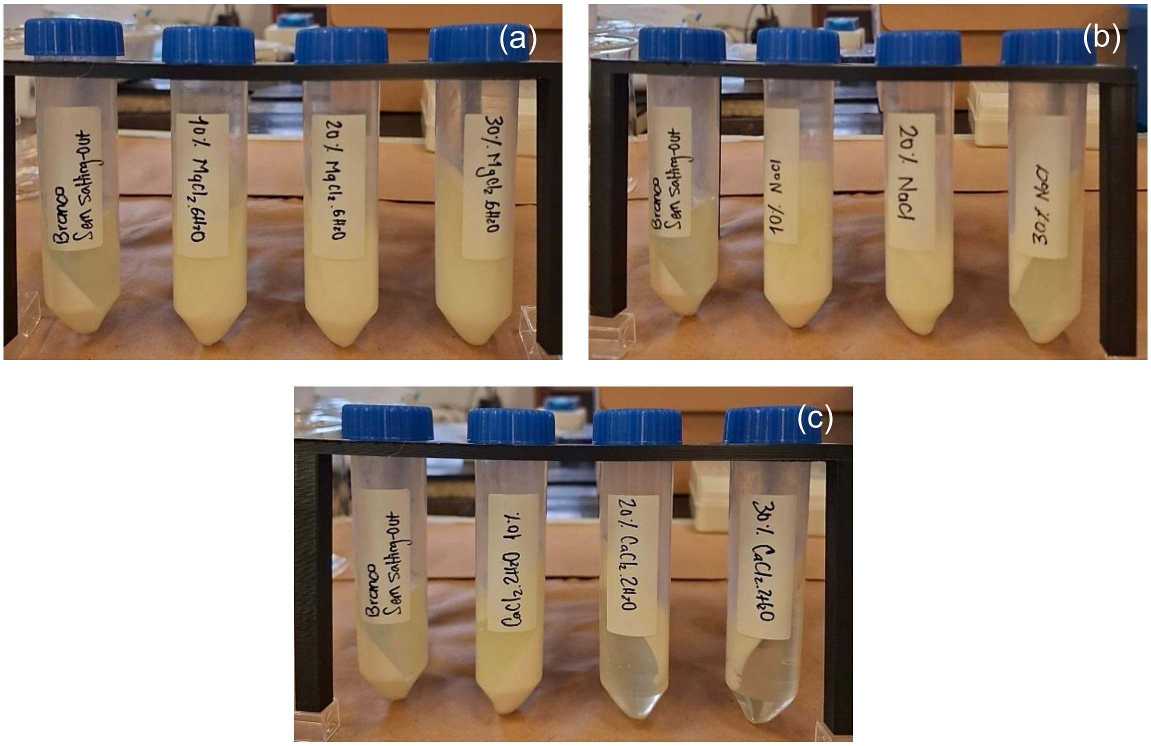

Figure S4- Thermogravimetric (TG) and differential thermal analysis (DTA) curves of **(a)** urea$_s$, **(b)** $CaCl_2.2H_2O_s$, **(c)** Urea in aqueous solution (20 mg mL^{-1}), and **(d)** Urea in serum milk (20 mg mL^{-1}).

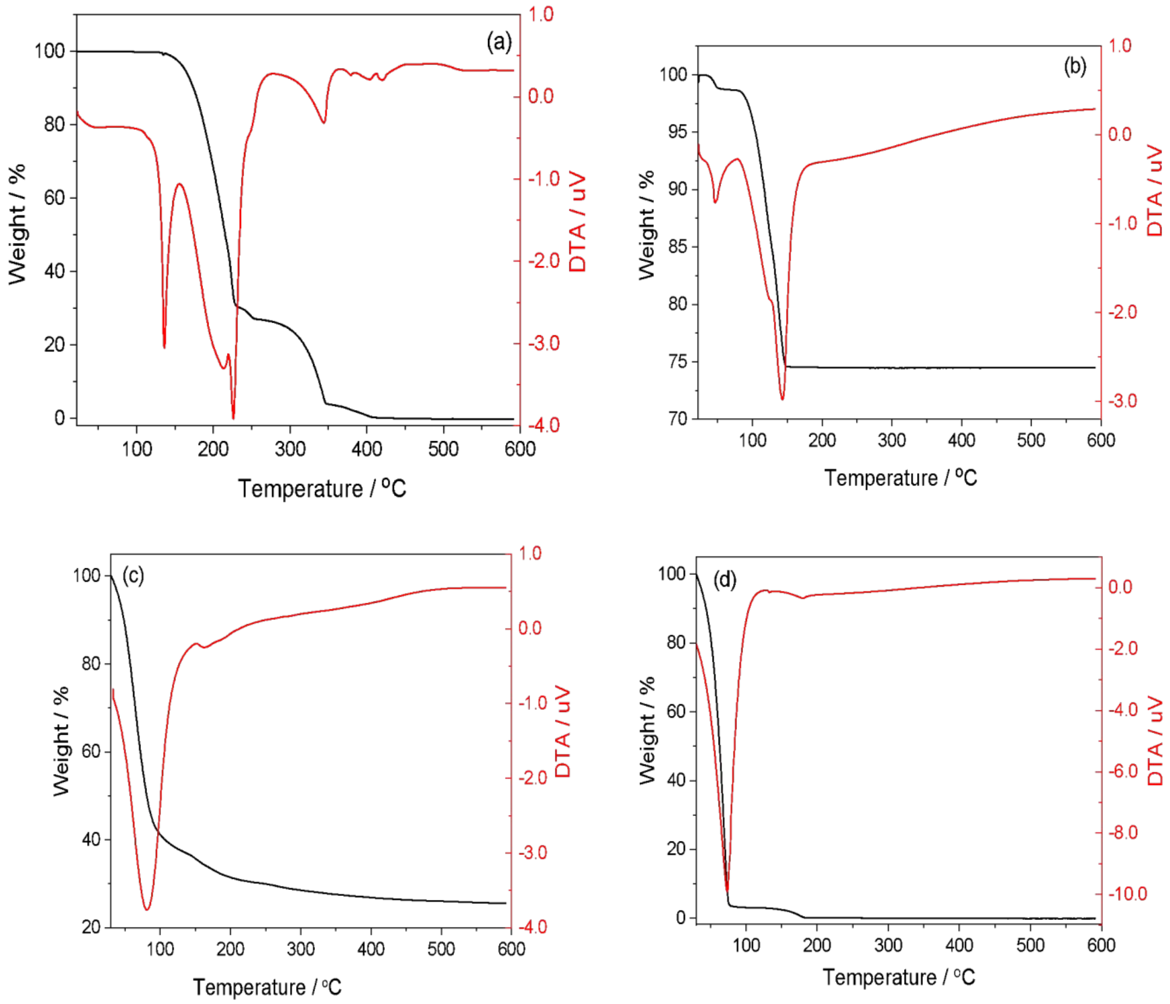

Figure S5-(a) Kinetic reaction of p-DAC with urea obtained by the UV spectrophotometer **(b)** Different procedures for adding solid urea or solution before and after precipitation. *Experimental conditions:* 30 mg mL^{-1} and 8.0 mg mL^{-1} of urea, 500 mg mL^{-1} in solution; 30% $CaCl_2.2H_2O$, HCl (6.0 mol L^{-1}), 3.0 % *p*-DAC (EtOH/ 0.5 mol L^{-1}), 70 μL, and 530 nm of absorbance in whole milk (3.0% fat).

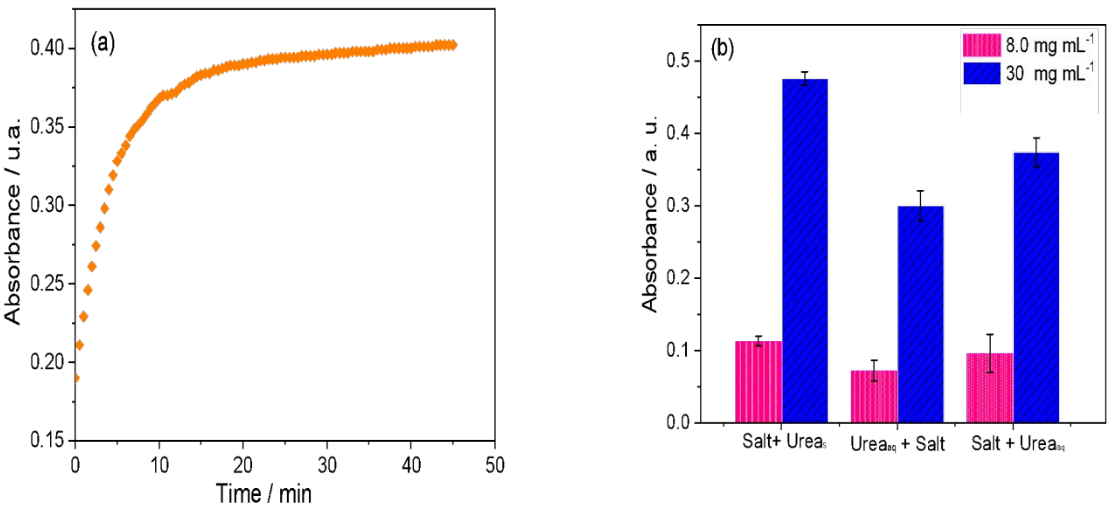

Figure S6-(a) Full scan mass spectrum for the solution containing Urea and *p*-DAC in an acid medium**.**
(b) Fragmentation mass spectrum (MS/MS) of the proposed reaction product between urea and *p*-DAC.

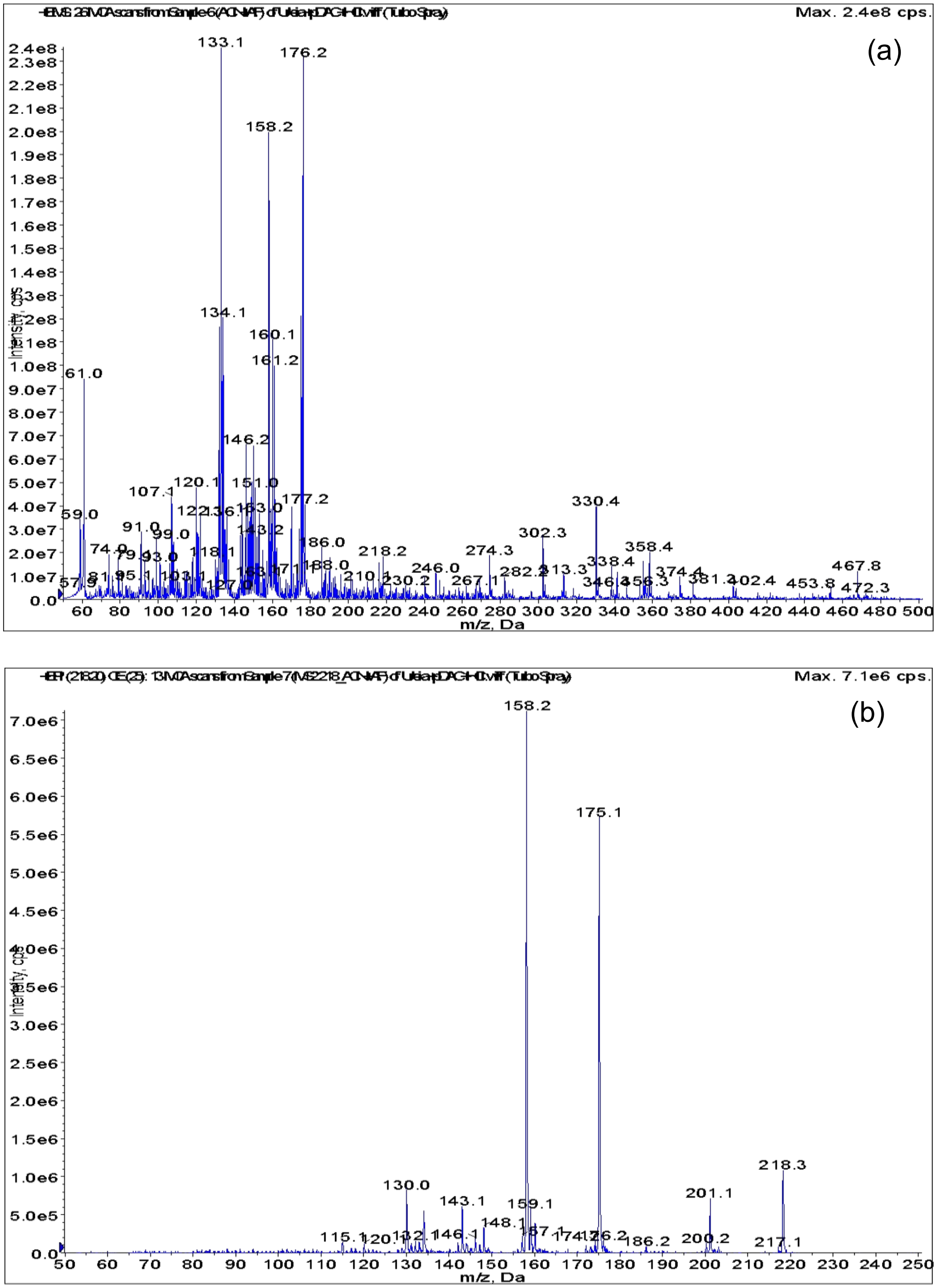

Figure S7- Fragmentation pathway for the precursor ion *m/z* 218 using collision-induced dissociation.

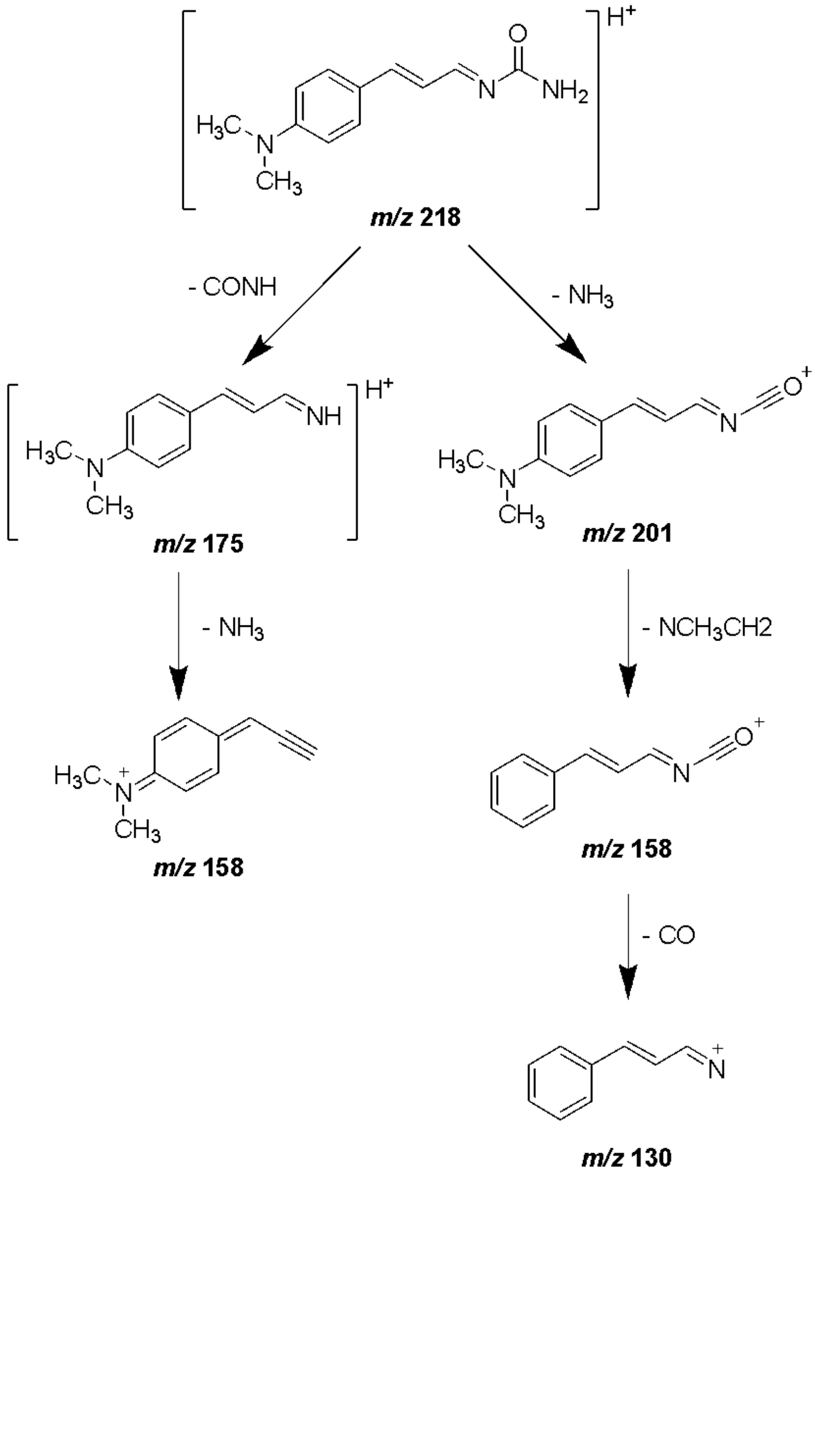

Figure S8- Photography of the colorimetric reaction between urea and *p*-DAC 3.0% w/v in 0.5 mol L^{-1} HCl/ ethanol.

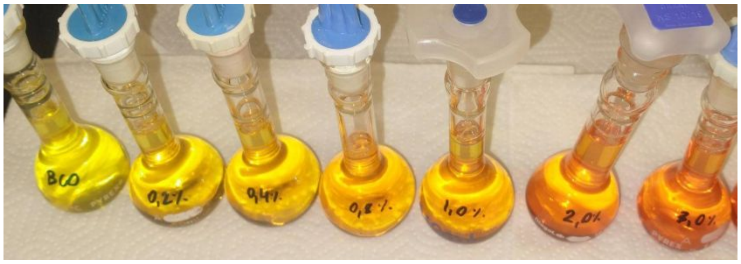

Table S3- Determination of figures of merit for urea quantification using HPLC-UV-Vis in whole milk (3.0% fat)

Milk sample	Methods Analytical	LOQ Urea (mg mL^{-1})	Calibration curve	R^2	Mean spike recovery ± %RSD		Intra-day precision (%RSD)	Inter-day precision (%RSD)
					Rec $_{medium}$	Rec $_{High}$		
**Whole milk (3.0% fat)**	**HPLC-UV-Vis**	3.0	y = 657.1X + 233.1	0.988	93.8 ± 5.65	91.8 ± 5.20	0.96	1.43

Table S4- Statistical analysis ANOVA, t-test, and F-test, comparison between spectrophotometer, 3D-printed photometer, and HPLC-UV-Vis in whole (3.0 % fat) and skim (0.5 % fat) milk samples.

Milk sample	Whole milk (3.0 % w/v fat)			Skim milk (0.5% w/v fat)	
Equipment / Parameter	Spectrophotometer	3D-printed photometer	HPLC-UV-Vis	Spectrophotometer	3D-printed photometer
$F_{tabulated}$	2012	250	558	372	408
$F_{meaning}$	9.62×10^{-21}	3.47×10^{-11}	7.23×10^{-14}	1.68×10^{-12}	8.14×10^{-13}
Degrees of freedom	20	18	17	17	17
Standard error	0.024	0.046	1370	0.072	0.042

	3D-printed photometer/ spectrophotometer	3D-printed photometer/ HPLC-UV-Vis	3D-printed photometer/ spectrophotometer
F-test	$p= 0.17 > 0.05$	$p = 0.39 > 0.05$	$p= 0.42 > 0.05$
t-test	$p_b = 0.81 > 0.05$	$p_b = 0.63 > 0.05$	$p_b=0.97 > 0.05$
